# Critical review of the phytohemagglutinin assay for assessing amphibian immunity

**DOI:** 10.1093/conphys/coad090

**Published:** 2023-12-12

**Authors:** Lauren Hawley, Kelly L Smalling, Scott Glaberman

**Affiliations:** Department of Environmental Science and Policy, George Mason University, Fairfax, VA, USA; New Jersey Water Science Center, U.S. Geological Survey, Lawrenceville, NJ, USA; Department of Environmental Science and Policy, George Mason University, Fairfax, VA, USA

**Keywords:** Amphibians, assay, immunology

## Abstract

Infectious diseases are a major driver of the global amphibian decline. In addition, many factors, including genetics, stress, pollution, and climate change can influence the response to pathogens. Therefore, it is important to be able to evaluate amphibian immunity in the laboratory and in the field. The phytohemagglutinin (PHA) assay is an inexpensive and relatively non-invasive tool that has been used extensively to assess immunocompetence, especially in birds, and more recently in amphibians. However, there is substantial variation in experimental methodology among amphibian PHA studies in terms of species and life stages, PHA doses and injection sites, and use of experimental controls. Here, we compile and compare all known PHA studies in amphibians to identify knowledge gaps and develop best practices for future work. We found that research has only been conducted on a limited number of species, which may not reflect the diversity of amphibians. There is also a lack of validation studies in most species, so that doses and timing of PHA injection and subsequent swelling measurements may not effectively evaluate immunocompetence. Based on these and other findings, we put forward a set of recommendations to make future PHA studies more consistent and improve the ability to utilize this assay in wild populations, where immune surveillance is greatly needed.

## Teaser

Infectious diseases are contributing to global amphibian declines. Scientists need better immune tests to identify vulnerable amphibian populations. This study reviews one such test in amphibians called the phytohemagglutinin (PHA) assay. The authors identify knowledge gaps and recommend standardized procedures to enhance the PHA assay’s effectiveness for conservation efforts.

## Introduction

In an era of unprecedented biodiversity loss, the ongoing global decline of amphibians is among the most severe (Bishop *et al.*, 2012). Forty percent of amphibian species are threatened and 70% are in decline—a downward trend that is expected to continue in the coming decades (Collins and Halliday, 2005; Hayes *et al.*, 2010; Bolochio *et al.*, 2020). Infectious disease has been repeatedly identified as a primary stressor (Vredenburg *et al.*, 2010; Bienentreu and Lesbarrères, 2020). There are a variety of pathogens in amphibians, including bacteria such as *Aeromonas hydrophila*, viruses such as Ranavirus, fungi such as *Batrachochytrium dendrobatidis* (*Bd*), and macroparasites such as *Ribeiroia* spp., that have impacted amphibian populations (Mauel *et al.*, 2002; Daszak *et al.*, 2003). Certain pathogens, notably *Bd* and Ranavirus, have been directly implicated in the mortality, decline, extirpation, and even extinction of amphibian species (Fernández-Benéitez *et al.*, 2008; Vredenburg *et al.*, 2010; Miller *et al.*, 2011; Scheele *et al.*, 2019). Chytridiomycosis represents what some experts believe is the greatest disease-driven extinction event in vertebrate history (Skerratt *et al.*, 2007; Vredenburg *et al.*, 2010).

Previous research indicates that amphibians vary in their ability to defend against pathogens (Knapp *et al.*, 2016; Lips, 2016; Scheele *et al.*, 2019). Genetics, body size and microbiota all play roles in disease susceptibility (Lips, 2016). At the same time, external factors, such as climate change (Rohr and Raffel 2010; Brunner *et al.*, 2015), habitat fragmentation (Greer and Collins, 2008) and pollution (Forson and Storfer, 2006; Brown *et al.*, 2021), impact the immune response to disease in complex ways (Hayes *et al.*, 2010; North *et al.*, 2015; Rollins-Smith, 2017; Ohmer *et al.*, 2020). For instance, a study by Rohr and Raffel (2010) suggests that increased climatic variability can suppress amphibian immune systems as they ‘lag behind’ rapid changes in temperature, and may simultaneously increase pathogen abundance by inducing *Bd* spore release. Habitat fragmentation, meanwhile, may alter disease dynamics via changes in host density (Greer and Collins, 2008), limitations to host gene flow and stress caused by factors such as predator density (North *et al.*, 2015). Contaminants may cause direct immune suppression via disruption of genetic and chemical processes and direct destruction of cell membranes (Hayes *et al.*, 2010), or indirect immunosuppression by causing changes in behavior and development, which increase the probability of exposure to pathogens (Rohr *et al.*, 2013) or reducing energy available for the immune response (Rohr *et al.*, 2008; Langerveld *et al.*, 2009). The relationship between environmental factors, immune system function and disease outcomes—whether an infection develops, the severity of infection and mortality rate—is vital to understanding and managing amphibian declines (Lips, 2016; Scheele *et al.*, 2019). Therefore, the ability to assess immunocompetence is critical for determining which individuals or populations are most at risk from disease and other environmental stressors (Young *et al.*, 2001; Collins, 2010; Blaustein *et al.*, 2012). For the purposes of this paper, immunocompetence refers to the ability of an individual’s immune system to respond to and defend against pathogens or other foreign substances. Martin *et al.* (2006) discusses the different components of the immune system and the importance of considering the broader ecological context when studying immune function. They also suggest that measuring a set of immune characteristics can provide a more nuanced understanding of an individual’s immunocompetence, since no test can evaluate every aspect of a vertebrate immune system.

While measuring amphibian immunity is essential for identifying at-risk populations, there are few immunological tools available for this purpose (Clulow *et al.*, 2015). Most methods, including leukocyte counts, complement protein quantification tests, bacterial-killing assays and immunoglobulin levels, require collecting blood samples (Christin *et al.*, 2003; Demas *et al.*, 2011; Priyadarshani *et al.*, 2015; Goetz *et al.*, 2017; Rodriguez and Voyles, 2020), which is technically challenging and can be harmful to amphibians due to their small size and limited venipuncture sites (Heatley and Johnson, 2009). Cardiac puncture is often used to obtain adequate blood volume, despite substantial risk to the animals (Heatley and Johnson, 2009). Other obstacles to applying most immune testing tools to amphibians include the need for rapid transportation of samples to the lab for processing (e.g. macrophage phagocytic ability assays), expensive equipment (e.g. natural killer cell toxicity assays), species-specific reagents (e.g. cytokine profiles), sterile conditions (e.g. lymphocyte proliferation assays) and/or extensive training (e.g. anti-microbial peptide analysis) (Christin *et al.*, 2003; Mangoni, 2006; Demas *et al.*, 2011; Clulow *et al.*, 2015; Goetz *et al.*, 2017). In addition to a lack of appropriate immunological tools, there are also few well-studied amphibian species (Carey *et al.*, 1999). Several species such as *Xenopus laevis*, *X. tropicalis*, *Rana pipiens* and *Ambystoma mexicanum* have been extensively used as immunological models, mainly because they are easy to breed and/or maintain in the laboratory (Womack *et al.*, 2022). However, these model species only represent a small fraction of the 53 described amphibian families, and do not necessarily represent the full range of amphibian immune system structure or function (Rollins-Smith and Woodhams, 2011; Levy and Heald, 2016; Lips, 2016; Bolochio *et al.*, 2020).

The phytohemagglutinin (PHA) assay is a method for assessing the immune response that avoids the limitations of other immune assays, as it is affordable, technically straightforward and does not require a blood sample (Martin *et al.*, 2006). PHA is an extract derived from red kidney beans *(Phaseolus vulgaris)*, which stimulates the innate immune system through inflammation and cell-mediated adaptive immune response with the recruitment of T cells and other leukocytes (Martin *et al.*, 2006; Salaberria *et al.*, 2013). This process induces a swelling response that is measured and converted into an index of immunocompetence (Tella *et al.*, 2008; Al-Khalaifah and Al-Nasser, 2020). While the PHA assay is primarily considered a test of adaptive immunity, cells associated with the innate immune system also play a vital role in swelling, particularly in the initial response, and antigen-presenting cells are not needed to stimulate the T-cell response (Martin *et al.*, 2006; Vinkler *et al.*, 2010). Thus, the PHA assay provides a complex demonstration of both innate and adaptive immune responses, making it a particularly valuable test for evaluating immunocompetence (Tella *et al.*, 2008; Vinkler *et al.*, 2010).

The PHA assay is affordable, relatively non-invasive and has been used extensively over the last 5 decades (Martin *et al.*, 2006; Tella *et al.*, 2008). Studies in birds have associated PHA responses with a range of other measures, including contaminant levels, noise pollution and coloration (Grasman *et al.*, 1996; Smits and Williams, 1999; Møller and Cassey, 2004; Møller and Saino, 2004; Moreno *et al.*, 2005; Grasman, 2010; Sivaraman and Kumar, 2013; Lopez-Antia *et al.*, 2015; Kerimov *et al.*, 2018; Pusch and Navara, 2018; Arct *et al.*, 2019; Minias *et al.*, 2019; Obomsawin *et al.*, 2021), and have assessed the impact of immunocompetence on health outcomes (Mougeot *et al.*, 2004; Moreno *et al.*, 2005; Sivaraman and Kumar, 2013; Martínez *et al.*, 2018). While the PHA assay is still often used for avian studies (O’Connor *et al.*, 2014; LaMonica *et al.*, 2021), it has also grown increasingly popular among other taxa, including mammals, reptiles and fish (Finger *et al.*, 2015; Hernández-Arciga *et al.*, 2018; Merlo *et al.*, 2018; Plasman *et al.*, 2019; Nasri *et al.*, 2020; LaMonica *et al.*, 2021; Stienbarger *et al.*, 2021; Miyazawa *et al.*, 2022).

The use of PHA assay in amphibians is relatively recent, with substantially fewer studies compared with birds. Published amphibian studies vary widely in terms of species, methodology (e.g. dosage), experimental design (e.g. controls) and results (e.g. swelling response), making it challenging to develop consistent best practices across a range of amphibian species. Moreover, most amphibian PHA studies have been conducted under controlled laboratory conditions, and no formal protocol has been developed for wild populations. To date, there has never been a critical review of PHA testing in amphibians to evaluate previous studies and highlight future needs. Our review fills this gap with three main objectives: (1) capture variation in both organismal and experimental aspects of published amphibian PHA studies; (2) identify uncertainties or gaps in the overall literature that limit the use of the PHA assay in amphibians; (3) propose future research directions to fill these gaps, with the goal of utilizing the PHA assay with amphibians under field conditions. It is our hope that this review will improve the use of the PHA assay for assessing the effects of disease and co-stressors on amphibian populations, which will benefit amphibian conservation.

## Literature Search & Data Collection

All amphibian PHA studies were compiled through a systematic literature search using the Preferred Reporting Items for Systematic Reviews and Meta-Analysis (PRISMA) statement (Moher *et al.*, 2009; Page *et al.*, 2021). PRISMA provides a set of criteria for carrying out and reporting the outcome of systematic reviews (Sarkis-Onofre *et al.*, 2021). Full details of the literature search process and keywords using PRISMA are provided (Supplementary Fig. S1). After capturing all known amphibian PHA studies, we extracted the following types of data from each study for further review: author(s), publication year, study aim, species, source of specimens, sample size, inoculation, control design, injection site, PHA concentration and injection volume, swelling response, and experimental time points (Table 1). We focused on these data types because they allow comparison of methodologies across studies and species.

**Table 1:** Summary of amphibian PHA assay studies

Study	Species	Average adult weight^a^ (g)	Average adult SVL^a^ (mm)	Life stage	Injection site	Conc. (mg/ml)	Injected volume (ml)	PHA per injection (g)	Times measured	Inoculation	PHA control type	Specimen source	Sex tested
Abu Bakar *et al.*, 2016	*L. aurea*	50	93.9	Juvenile, adult	Subcutaneous anterior lateral surface lower leg below knee	1	0.04	0.04	35 h	No	Bilateral	Captive bred	F, M
Bakewell *et al.*, 2021	*A. punctatus*	60	76	Adult	Subcutaneous anterior lateral surface lower leg below knee	2	0.05	0.1	24 h	No	Bilateral	Field collected from a large area	F, M
Brannelly *et al.*, 2019	*L. pipiens*	39.2	165	Juvenile	Hind thigh	25	0.02	0.5	12, 24, 48 h	Yes	Bilateral	Egg masses from field	F, M
Brown and Shine, 2014	*R. marina*	106.25	241	Adult	Toe web between 2nd and 3rd digits in hind foot	2	0.05	0.1	6, 12, 24, 48, 72 h	No	Bilateral	Field collected from 1 site	F, M
Brown *et al.*, 2011	*R. marina*	106.25	241	Adult	Toe web between 2nd and 3rd digits in hind foot	2.67	0.05	0.13	6, 12, 24, 48, 72 h	Subset	Bilateral	Field collected from 1 site	F, M
Brown *et al.*, 2015	*R. marina*	106.25	241	Adult	Toe web between 2nd and 3rd digits in hind foot	2	0.05	0.1	24, 48, 72 h	No	Bilateral	Field collected from 1 site	F, M
Cassettari *et al.*, 2022	*Rhinella granulosa*	—	90	Adult	Subcutaneous hind fleshy base of foot	20	0.01	0.2	6, 12, 24 h	No	Bilateral	Field collected from 1 site	F, M
Ceccato *et al.*, 2016	*L. peronii*	12.17	72.8	Larvae, juvenile	Right side of tail OR hind thigh	12	0	0.05	24, 48 h	No	None	Egg masses from field	F, M
Clulow *et al.*, 2015	*L. aurea*	50	93.9	Juvenile, adult	Subcutaneous anterior lateral surface lower leg below knee	1	0.04	0.04	6, 12, 24, 48 h	No	Bilateral	Varied field collected and bred in captivity	F, M
	*L. revelata*	0.93	35							No			
	*L. peronii*	12.17	72.8							No			
	*L. tasmaniensis*	3.37	50.5							No			
de Assis *et al.*, 2015	*R. icterica*	120	190	Adult	Subcutaneous hind fleshy base of foot	20	0.01	0.2	12 h	No	Bilateral	Field collected from 1 site	M
Desprat *et al.*, 2015	*H. arborea*	9.5	50	Adult	Right hind leg	25	0.02	0.5	18 h	No	Bilateral	Field collected from 1 site	M
Desprat *et al.*, 2017	*H. arborea*	9.5	50	Adult	Leg	25	0.02	0.5	18 h	No	Both	Field collected from 1 site	M
Fites *et al.*, 2014	*X. laevis*	130	147	Adult	Intramuscular middle plantar side hind foot	2	0.1	0.2	24, 48 h	Yes	Bilateral	Purchased from vendor	F
Gervasi and Foufopoulos, 2008	*Lithobates sylvatica*	17.6	83	Juvenile	Subcutaneous ventral hind thigh	1	0.05	0.05	25, 48 h	No	None	Egg masses from field	F, M
Gilbertson *et al.*, 2003	*L. pipiens*	39.2	165	Adult	Middle toe hind foot where webbing ends	2	0.06	0.13	24, 48, 72 h	Yes	Bilateral	Field collected from different sites	F, M
Iglesias-Carrasco *et al.*, 2018	*P. perezi*	31.8	86	Adult	Hind footpad	2	0.01	0.02	24 h	No	None	Field collected from 4 sites	M
Josserand *et al.*, 2015	*H. arborea*	9.5	50	Adult	Hind leg muscle	25	0.02	0.5	12, 14, 16, 18, 20, 22, 24 h	Subset	Mixed	Field collected from 1 site	M
Madelaire *et al.*, 2017	*Rhinella jimi*	—	—	Adult	Hind fleshy base of foot	20	0.01	0.2	12, 24 h	No	Bilateral	Field collected from 1 site	M
	*R. granulosa*	—	90							No			
	*Pleurodema diplolister*	—	20							No			
Madelaire *et al.*, 2019	*R. jimi*	—	—	Adult	Hind fleshy base of foot	20	0.01	0.2	12, 24 h	No	Bilateral	Field collected from 1 site	F, M
	*R. granulosa*	—	90							No			
	*P. diplolister*	—	20							No			
Murillo-Rincón *et al.*, 2017	*Rana arvalis*	10.8	—	Llarvae	Between 4th and 5th muscular segment of tail	1000	0.02	20	48 h	No	Whole specimen	Captive bred from field-collected frogs	F, M
Seiter, 2009	*L. sylvatica*	17.6	83	Larvae	Subcutaneous base of tail	2	0.02	0.03	24, 48 h	No	None	Egg masses from field	F, M
Szuroczki *et al.*, 2016	*L. pipiens*	39.2	165	Larvae	Tail	1	0.01	0.01	24, 48 h	No	Whole specimen	Purchased from vendor	F, M
Szuroczki *et al.*, 2019	*L. sylvatica*	17.6	83	Larvae	Tail	1	0.01	0.01	24 h	No	Whole specimen	Egg masses from field	F, M
Titon *et al.*, 2016	*Hypsiboas albopunctatus*	75	40	Adult	Subcutaneous hind fleshy base of foot	20	0.01	0.2	12, 24 h	No	Bilateral	Field collected from 1 site	F, M
Titon *et al.*, 2019	*R. ornata*	12.1	84.29	Adult	Hind fleshy base of foot	20	0.01	0.2	12, 24 h	No	Bilateral	Field collected from 1 site	M
Titon *et al.*, 2021	*R. icterica*	120	190	Adult	Hind fleshy base of foot	20	0.01	0.2	12 h	No	Bilateral	Field captured	M
Troïanowski *et al.*, 2017	*H. arborea*	9.5	50	Adult	Hind leg	50	0.02	1	16 h	No	Bilateral	Field collected from 1 site	M
Vasconcelos-Teixeira *et al.*, 2022	*Rhinella diptycha*	—	—	Adult	Hind fleshy base of foot	20	0.01	0.2	12, 18 h	No	Whole specimen	Field collected from 3 sites	F, M
Venesky and Laskey, 2022	*P. cinereus*	65	412	Adult	Tail	4	0.01	0.04	24 h	No	None	Field collected from 1 site	F, M
Venesky *et al.*, 2012	*Lithobates sphenocephalus*	65.7	127	Larvae	2 mm from tip of tail, right side, at junction of tail and muscle	1	0.02	0.02	48 h	No	Bilateral	Egg masses from field	F, M
Wang *et al.*, 2019	*X. laevis*	130	147	adult	middle plantar side of foot – random hind/fore	2	0.1	0.2	24 h	No	Bilateral	Purchased from vendor	M
Young *et al.*, 2014 ^b^	*Litoria caerulea*	50	100	Adult	Toe web between 2nd and 3rd digits in hind foot	5	0.1	0.5	6, 12, 24, 48 h	No	None	Field collected from different sites	F, M
	*Litoria infrafrenata*	24.46	110							No			
Zamora-Camacho, 2019	*E. calamita*	62.7	100	Adult	Subcutaneous forelimb sole pad	100	0.01	1	1, 2, 3, 4, 5, 6 h	No	Bilateral	Field collected from 2 sites	F, M
Zamora-Camacho *et al.*, 2022	*P. perezi*	31.8	86	Adult	Hind fleshy base of foot	10	0.01	0.1	24 h	No	None	Egg masses from field	F, M
Zamora-Camacho and Comas, 2018	*E. calamita*	62.7	100	Adult	Subcutaneous forelimb sole pad	10	0.01	0.1	1, 2, 3, 4, 5, 6 h	No	Bilateral	Field collected from 1 larger area	F, M
Zhang *et al.*, 2017	*P. nigromaculatus*	56.4	90.8	Adult	Toe web between 2nd and 3rd digits in hind foot	3.3	0.05	0.17	6, 12, 24, 48, 72 h	No	Bilateral	Field collected from 1 larger area	F, M
	*P. raddei*	—	—							No			
	*B. gargarizans*	116.2	134							No			

a ^a^Data are averages for adult of the species based on AmphBIO (Oliveira *et al.*, 2017).

b This was the only study in our review that did not detect a positive response.

**Figure 1:**
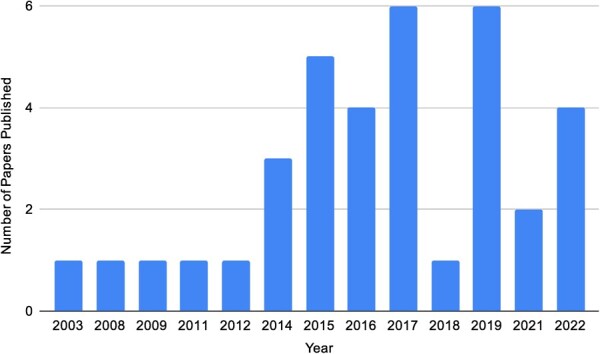
Timeline of published PHA studies in amphibians. Search was performed according to PRISMA (see Supplementary Fig S1 for full search details).

We found a total of 36 amphibian studies that have utilized the PHA assay. The first was published in 2003, and studies continued sporadically thereafter until 2014, at which point publications began to increase (Fig. 1).

## Analysis of Existing Amphibian PHA Studies

We divided our critical review of amphibian PHA studies into two sections: ‘Organismal Variables’, which focuses on multiple attributes of organisms used for testing (e.g. species, sex, life stage), and ‘Experimental Variables’, which focuses on aspects of experimental study design (e.g. PHA injection site and concentration). All information collected from amphibian PHA studies is summarized in Table 1.

### Organismal Variables

#### Species

**Figure 2:**
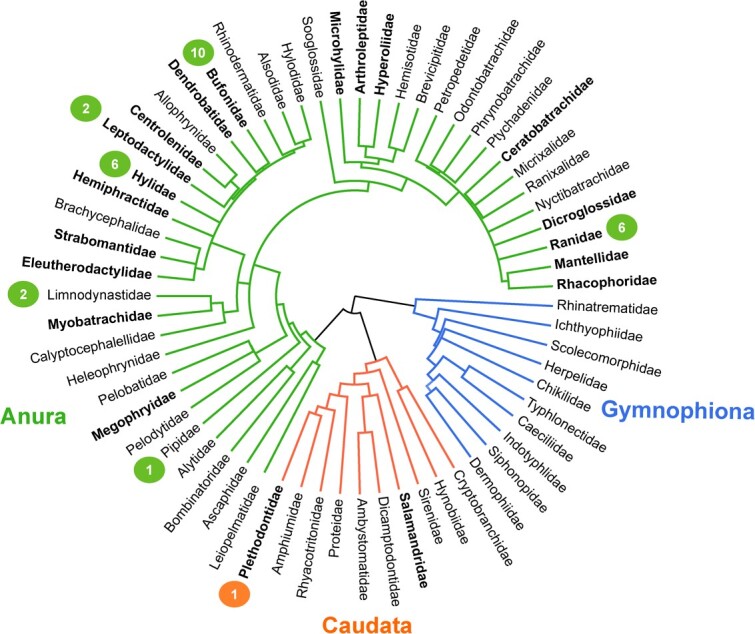
Amphibian phylogenetic tree by taxonomic family. Families in bold contain >100 species according to AmphibiaWeb (https://amphibiaweb.org). Families in italics contain >10 species listed as critically endangered by the IUCN Red List. Circles represent the number of species within a given family that have been tested in PHA studies (see Table 1). The phylogenetic tree was generated using all available amphibian taxa in TimeTree (http://www.timetree.org)

Out of 36 PHA studies in amphibians, a total of 27 distinct species were examined representing seven different taxonomic families (Fig. 2; Table 1). All publications focused on frogs, except for a single study on salamanders, specifically *Plethodon cinereus* (Venesky and Laskey, 2022), and there have been no studies on caecilians. As expected, species-rich anuran families such as Ranidae (aquatic frogs), Hylidae (treefrogs and relatives), and Bufonidae (toads) are well represented. However, there are many large amphibian families or clades with substantial numbers of species that are not represented in PHA studies (Fig. 2), such as the Microhylidae, generally smaller, narrow-mouthed frogs, which contains >700 species.

Interspecific variation has been observed in swelling response (Clulow *et al.*, 2015; Zhang *et al.*, 2017) and lymphocyte proliferation (Wiley and Meisner, 1984). Therefore, analyzing a wide range of species is particularly important for the PHA assay. Variation in assay results, accounting for variation in sex and body size, has been found even among closely related species such as *Limnodynastes peronii* and *Limnodynastes tasmaniensis* (Clulow *et al.*, 2015). Experts have cautioned against interspecific comparisons of PHA results (Martin *et al.*, 2006), in part, due to varied investment in innate immunity among taxa (Woodhams *et al.*, 2006). Martin. *et al.* suggests, based on much more robust avian literature, that these differences are due to a combination of the variety of immune cells involved in PHA oedema and variation in investment in production of different immune cells among species to support different environments, life histories and physiology (2006).

The species tested in PHA studies are geographically unrepresentative as well. Species from Australia, China and North America are over-represented, while species from Africa and South America are substantially under-represented (Supplementary Fig. S2). This is problematic, not only because certain geographically limited taxa remain under-represented, but also because different regions have diverse ecological conditions that make it challenging to extrapolate results from species in other areas (Christie *et al.*, 2020). Studying these species in a laboratory setting is a vital first step in investigating these differences and establishing standardized methods that are effective in diverse geographic areas. Both taxonomic and geographic biases are known problems for amphibian research (Christie *et al.*, 2021). There is a clear imperative to test and/or extrapolate results to more species in underrepresented taxonomic groups and global regions. To further this goal, there are multiple species from understudied areas and taxa with known husbandry information (e.g. dendrobatid frogs) that could be used in future studies.

#### Life stage and age

Life stage, metamorphosis and age have complex effects on immune function and disease outcomes (Flajnik *et al.*, 1987; Rollins-Smith, 1998; Haislip *et al.*, 2011; Grogan *et al.*, 2018). All PHA studies conducted so far have either tested a single life stage or placed alternative life stages into different experimental groups to avoid any confounding effects of age on results (Clulow *et al.*, 2015). Most studies have been performed on adults (Table 1), although several studies have been performed on larvae (Venesky *et al.*, 2012; Ceccato *et al.*, 2016; Szuroczki *et al.*, 2016, 2019; Murillo-Rincón *et al.*, 2017; Venesky and Laskey, 2022) or juveniles (Clulow *et al.*, 2015; Abu Bakar *et al.*, 2016; Ceccato *et al.*, 2016).

Only two studies have directly compared age and swelling responses from PHA, while two others compared development time and PHA response (Abu Bakar *et al.*, 2016; Zamora-Camacho and Comas, 2018). Abu Bakar *et al.* (2016) found that PHA-induced oedema in *Litoria aurea* exposed to *Bd* was higher in adults than in either subadults or juveniles. They also found that while *Bd* exposure reduced PHA-related swelling for all groups, the reduction was less pronounced in adults (Abu Bakar *et al.*, 2016). The authors suggested that this response may illustrate maturation and recovery of the adaptive immune response after metamorphosis. In general, larval immune systems are less developed and robust than adult immune systems, and aspects of the immune system are suppressed during metamorphosis in anurans, although much less is known about urodeles (Rollins-Smith, 1998; Abu Bakar *et al.*, 2016; Grogan *et al.*, 2018). Another study on *Epidalea calamita* performed on adults exclusively found that swelling decreased with age, leading study authors to suggest that senescence may reduce immunocompetence after reaching adulthood (Zamora-Camacho and Comas, 2018). Two studies looking at the relationship between development time and immunocompetence found that increased development time in response to stress is associated with reduced PHA response (Gervasi and Foufopoulos, 2008; Seiter, 2009). Given the substantial remodeling of the amphibian immune system during metamorphosis, and the limited study across different age groups, further research in the laboratory is needed to elucidate the mechanism driving swelling-response variation between life stages (Flajnik *et al.*, 1987; Grogan *et al.*, 2018). Such study is an important precursor to field research since it would be impractical to limit field studies to similar-age individuals.

#### Sex

Variation in immunity among sexes is well known in vertebrates because of differences in physiology and life history traits (Nunn *et al.*, 2009). In amphibians, males and females can differ substantially in size, behavior and hormone levels (Shine, 1979; Licht *et al.*, 1983), all of which can impact immune function (French *et al.*, 2007; Taneja, 2018; Vitousek *et al.*, 2018). However, previous studies have found minimal differences in immunocompetence both among sexes and individuals of the same sex exhibiting variation in reproductive morphology and behavior during the mating season (Saad, 1992; Zapata *et al.*, 1992; Cornuau *et al.*, 2014; Thomas and Woodley, 2017). While some amphibian PHA studies looked at only males (de Assis *et al.*, 2015; Desprat *et al.*, 2015, 2017; Josserand *et al.*, 2015; Madelaire *et al.*, 2017; Troïanowski *et al.*, 2017; Iglesias-Carrasco *et al.*, 2018; Titon *et al.*, 2019, 2021; Wang *et al.*, 2019) or females (Fites *et al.*, 2014) to avoid confounding sex-related effects, several other studies specifically investigated sex-related differences in PHA swelling responses (Bakewell *et al.*, 2021; Zamora-Camacho *et al.*, 2022).

The results of amphibian studies investigating the influence of sex on PHA swelling have been inconsistent. A study of *Anaxyrus punctatus* reported greater swelling responses in females (Bakewell *et al.*, 2021), while another study with *E. calamita* showed earlier swelling in females but statistically indistinguishable peak swelling among sexes (Zamora-Camacho, 2019). A recent study of *Pelophylax perezi* found that larval exposure to ammonium impacted immunocompetence in adult males, including PHA-induced oedema at 24 hours, but not in adult females (Zamora-Camacho *et al.*, 2022). Several other studies with *Rhinella marina, L. aurea, Litoria revelata, L. peronii, L. tasmaniensis, Pelophylax nigromaculatus, Pseudepidalea raddei* and *Bufo gargarizans* showed no effect of sex on peak swelling (Brown and Shine, 2014; Brown *et al.*, 2015; Clulow *et al.*, 2015; Zhang *et al.*, 2017). Each of these studies accounted for variation in individual body size in drawing conclusions about the effect of sex, although they did not measure or evaluate any additional measures of sex, sex hormones or reproductive behavior. Overall, there is limited support that sex and reproductive activity influence PHA swelling response, but further study is warranted given the conflicting results and lack of species examined. Given the current uncertainty regarding these effects, studies should at the very least record sex and integrate it into PHA swelling analysis.

#### Body size

The PHA assay, like many immunological tools, is simpler to perform on larger specimens, because swelling is easier to measure and less likely to require precise equipment (Szuroczki *et al.*, 2016, 2019; Murillo-Rincón *et al.*, 2017). As a result, PHA studies have been biased towards medium or large animals, with nearly 90% of species studied being medium-sized or larger in adulthood (see Table 1). Studying species across the full spectrum of body sizes may be particularly important for amphibians, as there is evidence that smaller sizes are associated with increased vulnerability to declines (Cardillo, 2021). Fortunately, progress has been made to develop methodologies and tools appropriate for smaller specimens, including altered injection sites and the use of microscopy. See **Experimental Variables: PHA Injection** for more information on relevant methodology, as well as Clulow *et al.* (2015), Murillo-Rincón *et al.* (2017) and Szuroczki *et al.* (2016).

Body size variation within species may also affect PHA-related swelling, which experts have suggested may be due to either differing spleen size (Fites *et al.*, 2014; Vasconcelos-Teixeira *et al.*, 2022) or the higher cost of immune activation in smaller individuals (Venesky and Laskey, 2022). Six studies assessed the effect of conspecific body size on swelling response with mixed results: three studies reported no effect of body size on oedema (Brown and Shine, 2014; Brown *et al.*, 2015; Clulow *et al.*, 2015; Zamora-Camacho and Comas, 2018); two studies found that increased body size was associated with increased swelling (Brown *et al.*, 2015; Titon *et al.*, 2019); and one study found that decreased body size was associated with increased swelling (Bakewell *et al.*, 2021). Generally, increased body size would be expected to be associated with an increased immune response (Fites *et al.*, 2014; Vasconcelos-Teixeira *et al.*, 2022; Venesky and Laskey, 2022). The results of Bakewell *et al.* (2021) are therefore unexpected. Authors of this study suggest it is possible that results may have been a result of inaccurate measurements. Therefore, further study using more precise measurements (see **Experimental Variables: Swelling Response**) would be beneficial (Bakewell *et al.*, 2021). While many studies measure size using snout-vent length (SVL), measuring and reporting both SVL and weight of each individual tested would significantly improve future work by facilitating analysis of the impact of body size within and between studies.

### Experimental Variables

#### PHA injection

Injection procedures, including injection amount, site, and repetition, varied greatly among studies. Injection volumes of both PHA (diluted in saline solution) and PBS (control) varied from 4 μl (Ceccato *et al.*, 2016) to 100 μl (Fites *et al.*, 2014; Young *et al.*, 2014; Wang *et al.*, 2019). The most common injection volume of PHA or PBS solutions by far was 10 μl, which was used in 15 of 36 studies. The average injection volume across studies was approximately 30 μl. Given that injections of higher volumes can increase swelling regardless of content, maintaining an equivalent volume across all controls and treatments is important, as is minimizing injection volume when possible (Collins *et al.*, 2020).

The amount of PHA per injection ranged from 0.01 mg (Szuroczki *et al.*, 2016, 2019) to 20 mg (Murillo-Rincón *et al.*, 2017), with 0.2 mg being the most common (see Table 1). It should be noted that studies of large species such as *R. marina* and *Rhinella icterica* were successfully completed using a dose of only 0.1 mg, suggesting that higher doses may be unnecessary, and should be used only when lower doses are not successful (Brown *et al.*, 2011, 2015; Brown and Shine, 2014). Only two studies to date have attempted to establish an ideal dose of PHA for the assay. Brown *et al.* (2011) tested three doses: 0.05, 0.1 and 0.25 mg on large *R. marina*; while the PHA amount was not related to peak swelling response, swelling response time was slower at the lowest dose, with no difference between the 0.1- and 0.25-mg doses (Brown *et al.*, 2011). Josserand *et al.* (2015) found similar results comparing swelling responses of *Hyla arborea* injected with 0.25, 0.5 and 1 mg of PHA; while 0.25 mg was insufficient to generate a swelling response, there was no difference in peak swelling response between 0.5 and 1 mg. Based on these studies, it appears that once a minimum threshold is reached, increasing the PHA dose is unnecessary. Dosage also appears to vary substantially in avian studies (Smits *et al.*, 2002); however, a general protocol has been developed for birds by Grasman at a dose of 0.1 ml of 1 mg/ml PHA solution (Grasman, 2010).

**Figure 3:**
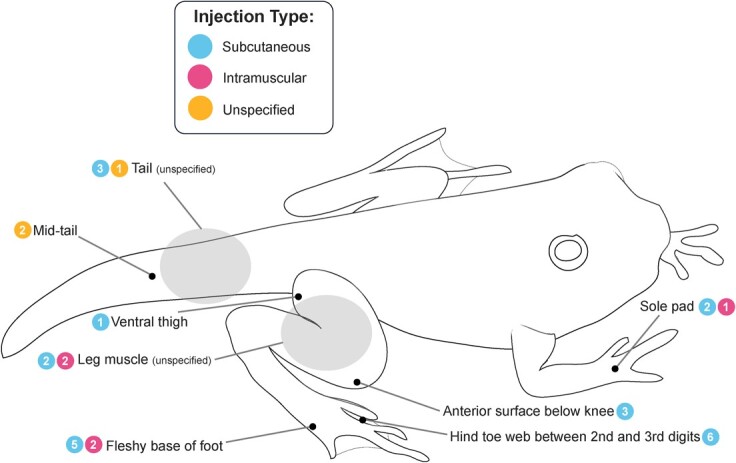
Anatomical map of PHA injection sites used across amphibian studies. Colors represent types of injection. Numbers inside circles show the number of studies utilizing each injection location. This froglet image represents an amalgam of different amphibian species and life stages since injections were performed on adult and larval frogs and salamanders.

A variety of injection sites were used across different PHA studies, species and life stages (Fig. 3). Injection sites should be tailored to the specimen type to generate an easily measurable swelling response (Clulow *et al.*, 2015). However, many studies do not provide an adequate description of the injection site for proper duplication, using unspecific terms such as ‘tail’, ‘leg’ and ‘hind leg’ (Szuroczki *et al.*, 2016; Desprat *et al.*, 2017; Troïanowski *et al.*, 2017). Still, some overall patterns can be observed. Subcutaneous injection of the toe web was often used for larger species but was not possible for smaller specimens or any specimens without toe webs (Clulow *et al.*, 2015; Josserand *et al.*, 2015). Methods used on smaller specimens included subcutaneous injections below the knee and intramuscular injections of the footpad and leg (Clulow *et al.*, 2015; Josserand *et al.*, 2015). The sole study that failed to generate a difference in swelling response between PHA and PBS controls used a subcutaneous toe web injection site (Young *et al.*, 2014). On the other hand, Clulow *et al.* (2015) found that upper leg injections were not as successful with larger species, while toe web injections were not successful with smaller species and proposed that the lower leg and foot pad are the two most broadly applicable methods for PHA. Some research found greater success with intramuscular injection (Fites *et al.*, 2014); however, there is histological evidence that PHA injections can cause muscle damage in some specimens, which contraindicates the use of intramuscular injection, unless necessary, until further research is conducted (Clulow *et al.*, 2015). One study randomly injected hind or forelimbs, introducing possible problems comparing results within the study (Wang *et al.*, 2019). Although avian studies were originally conducted using either the patagia or the foot as injection sites, there was no correlation found between swelling at these two sites (Kean and Lamont, 1994). Today, the patagia is used nearly universally (Martin *et al.*, 2006; Hasselquist, 2007; Grasman, 2010). It is important to evaluate and address the lack of comparability among injection sites in amphibian studies, although it is unlikely that a single universal injection site can be established. This issue should be thoroughly examined and taken into consideration when designing and interpreting amphibian studies.

For caudates and larval anurans, tail injections are easier because legs are either less prominent or absent altogether. For the smallest specimens, studies have adopted protocols with a high level of specificity of measurement to ensure comparability of swelling responses among individuals. Venesky *et al.* (2012), for instance, made injections and measurements 2 mm from the tip of the tail, while Murillo-Rincón *et al.* (2017) specified that injections and measurements took place between the fourth and fifth muscular segments of the tail. Such specificity is not found in publications of adult studies, although it would be beneficial.

Studies suggest that while the first PHA injection is a broad measure of immune response that engages both the innate and adaptive immune system, a second PHA injection elicits a more substantial adaptive immune response, prompting some researchers to use inoculation to provide a more robust measure of adaptive immunity (Fites *et al.*, 2014; Josserand *et al.*, 2015). Five of the 36 studies inoculated specimens with PHA in the weeks leading up to the assay. These studies, which included *Lithobates pipiens, R. marina, X. laevis* and *H. arborea*, all used inoculation to elicit a stronger measure of adaptive immunity. The only exception was Brown *et al.*’s study of *R. marina*(2011), which examined whether inoculation increased swelling response as would be expected if the adaptive immune response was enhanced by multiple exposures (2011). Their results showed that the second injection of inoculated specimens did cause increased swelling, supporting this hypothesis. A study by Fites *et al.* (2014) found that, while swelling response to the first PHA injection was not affected by exposure to *Bd*, swelling response to the second exposure was significantly impacted, suggesting the possibility that *Bd* supernatants affect the adaptive arm of the immune system more than the innate, though it should be noted that the adaptive and innate immune systems are not truly separate; in fact, the innate immune system initiates and guides the adaptive immune response (Dempsey *et al.*, 2003)*.* Still, inoculation certainly warrants further study, as it could facilitate assessment of more specific functions of the amphibian immune system. However, it should be noted that this method is not ideal for field use, as it would necessitate a much longer holding time of animals, which may be impractical.

#### Swelling response

Interstudy variation in swelling response is difficult to assess, because swelling has been reported differently among studies. Many studies used the approach adopted by Brown *et al.* (2015) and Zhang *et al.* (2017), reporting a proportion as change in thickness over starting thickness (Brown *et al.*, 2011, 2015; Brown and Shine, 2014; Clulow *et al.*, 2015; de Assis *et al.*, 2015; Abu Bakar *et al.*, 2016; Szuroczki *et al.*, 2016; Madelaire *et al.*, 2017, 2019; Murillo-Rincón *et al.*, 2017; Brannelly *et al.*, 2019; Titon *et al.*, 2019; Cassettari *et al.*, 2022; Vasconcelos-Teixeira *et al.*, 2022; Venesky and Laskey, 2022). Meanwhile, many other studies reported millimetres of swelling without providing a starting or ending thickness measure to allow comparison with percentage-based reporting (Gilbertson *et al.*, 2003; Gervasi and Foufopoulos, 2008; Seiter, 2009; Venesky *et al.*, 2012; Young *et al.*, 2014; Desprat *et al.*, 2015, 2017; Ceccato *et al.*, 2016; Szuroczki *et al.*, 2016, 2019; Titon *et al.*, 2016; Troïanowski *et al.*, 2017; Wang *et al.*, 2019; Zamora-Camacho, 2019; Zamora-Camacho *et al.*, 2022). Other methodologies have been used less often; for example, Zamora-Camacho and Comas (2018) calculated the ‘area under the curve’, by integrating swelling response measurements over multiple time points.

Swelling responses in PHA studies are measured at set times after injection using a variety of measurement equipment including digital calipers (Fites *et al.*, 2014; Wang *et al.*, 2019; Venesky and Laskey, 2022) and pressure-sensitive or adjustable force micrometers (Clulow *et al.*, 2015; Abu Bakar *et al.*, 2016; Zhang *et al.*, 2017; Zamora-Camacho and Comas, 2018) to measure relative width before and after swelling. While adjustable force micrometers are more accurate than digital calipers, they have also been tied to a ‘compression effect’, in which leg measurements decrease, or, in the treatment leg, increase less, due to compression of the tissue by the device. Compression effects should be accounted for via control measurements if these tools are used (Clulow *et al.*, 2015) (see **Experimental Variables: Experimental Controls**). However, a study using digital calipers also found negative swelling responses in some individuals, suggesting digital calipers should also be evaluated for compression effect (Ceccato *et al.*, 2016). Meanwhile, for smaller specimens, such as larvae, more specialized tools have been used. Murillo-Rincón *et al.* (2017) used photography, a reference grid and software to measure swelling, while Szuroczki *et al.* (2016, 2019) used a dissection microscope along with digital calipers. While the dissection microscope method requires anaesthesia (Szuroczki *et al.*, 2016, 2019), the photography and reference grid method appears to avoid the compression effect and reduce handling time without the need for anaesthesia; however, authors indicated that some results had to be excluded due to larval positioning in the photographs (Murillo-Rincón *et al.*, 2017). Therefore, while promising, a digital imaging protocol could be further developed in the future to ensure accuracy and feasibility among different species, life stages, body sizes and morphological features. While relatively expensive, magnetic resonance imaging (MRI) is still the best practice for digital imaging of soft tissues. However, advances in technology have allowed both analysis of 2D images, as in Murillo-Rincón *et al.* (2017), and more advanced photogrammetry, in which 2D images are used to generate a 3D model, though one less accurate than computed tomography (CT) scans (Ford *et al.*, 2023). Ford *et al.* (2023) provides an excellent overview of current methods of anatomical study, including the latest advances in digital imaging.

Measurement intervals, or time passed since injection when swelling measurements are taken, have differed among studies. Validation and optimization studies (i.e. studies designed to identify the timing of peak swelling) often involve taking many measurements at different time points, while immunology studies tend to choose one time point thought to coincide closely with peak swelling. Identifying the timing of peak swelling can be complex and varies considerably between species (Clulow *et al.*, 2015; Madelaire *et al.*, 2017, 2019; Zhang *et al.*, 2017; Zamora-Camacho, 2019), sex, and population (Zamora-Camacho, 2019). For example, Josserand *et al.* (2015) found that swelling occurred in *H. arborea* ~14 hours after injection, while Clulow *et al.* (2015) found that peak swelling occurred at 24 hours for *L. aurea*, *L. revelata* and *L. tasmaniensis,* but occurred at 48 hours for *L. peronii*. Therefore, Clulow *et al.* (2015) cautioned that, before the time frame for a given study is set, species should be validated, and measurement protocols optimized according to a standard protocol. Twenty-four hours is the most common time point used to measure peak swelling in amphibian PHA studies (Table 1). Many studies have used 24 hours as a default for species without carrying out a validation step to empirically determine peak swelling time (Iglesias-Carrasco *et al.*, 2018; Szuroczki *et al.*, 2019; Wang *et al.*, 2019; Bakewell *et al.*, 2021; Zamora-Camacho *et al.*, 2022). For avian studies, the standard protocol is to take measurements at ~24 hours (Grasman, 2010).

A similarly consistent time point that is effective across taxa for amphibians would facilitate future study, though validation of such a time point across taxa beforehand is certainly advisable. Although studies aim to capture the approximate time of peak swelling, which is expected to occur and subside within 72 hours after injection, there is no consensus on the optimal frequency of measurements required to achieve this objective. A ‘24-hour regime’, in which triplicate measures are taken at 6, 12, 24, 48 and 72 hours (Clulow *et al.*, 2015), was used in four methodology optimization studies (Brown *et al.*, 2011; Brown and Shine, 2014; Young *et al.*, 2014; Zhang *et al.*, 2017), while an adapted ‘every 4-hour regime’, in which single measurements are taken every 4 hours for the first 72 hours of the study, was only used by Clulow *et al.* (2015). These two optimization approaches have benefits and drawbacks; while more frequent 4-hour measurements would more accurately determine peak swelling, taking single rather than triplicate measurements could reduce precision and accuracy of the measurements themselves. Conversely, an increased number of measurements could increase handling stress and researcher effort, which must be weighed against the benefits of more thorough validation.

Nineteen studies, meanwhile, took measurements at two or fewer times, using species that had not been previously optimized with more granular measurements (Gervasi and Foufopoulos, 2008; Seiter, 2009; Fites *et al.*, 2014; de Assis *et al.*, 2015; Desprat *et al.*, 2015; Szuroczki *et al.*, 2016, 2019; Titon *et al.*, 2016, 2019, 2021; Madelaire *et al.*, 2017, 2019; Murillo-Rincón *et al.*, 2017; Troïanowski *et al.*, 2017; Iglesias-Carrasco *et al.*, 2018; Wang *et al.*, 2019; Bakewell *et al.*, 2021; Vasconcelos-Teixeira *et al.*, 2022; Zamora-Camacho *et al.*, 2022). Studies by Zamora-Camacho (2019) and Zamora-Camacho and Comas (2018) are unique in that they tested ‘early swelling peak’, within just 6 hours of injection. This approach is noteworthy as it could help improve practicality in the field by reducing waiting time. While innate immune response swelling occurs rapidly, before the combined innate and adaptive-driven peak (Szuroczki *et al.*, 2016), significant early swelling with injection of both PHA and PBS has been observed (Josserand *et al.*, 2015). Given that both of Zamora-Camacho, 2019 studies were performed without a control, replication of these studies with a PBS control would help verify whether this approach is appropriate.

The effects of PHA assay-induced swelling on amphibian mobility, health and stress are still uncertain. To date, no study has specifically sought to identify the post-assay effects of PHA itself on amphibians. One study by Iglesias-Carrasco *et al.* (2018) tested the effect of the PHA assay on hiding time and found no change from PHA treatment; however, this is an anti-predator behavior that relies relatively little on limb mobility. While a study of avian nestlings found that the PHA assay did not increase levels of stress proteins (Merino *et al.*, 1999), no similar study has been conducted on amphibians. Meanwhile, some studies suggest potential impacts of the PHA assay on amphibians that warrant further research. Zhang *et al.* (2017) noted that the PHA assay caused a reduction in body mass across the course of the study. Murillo-Rincón *et al.* (2017) found that when performed on larvae, the PHA assay increased the larval period, which is potentially problematic given that development time has been a long-standing measure of fitness and can impact survival in ephemeral environments. Desprat *et al.* (2017) observed that the PHA assay caused a lightening of the vocal sac in starved males proportional to the PHA response, which the authors suggested may be an indicator to females that a male recently encountered a disease agent, reducing chances of breeding success. Finally, histological analysis by Clulow *et al.* (2015) found evidence of damage to the muscle fibers and fibrosis in some specimens injected with PHA. While none of these findings preclude the use of the PHA assay for field studies, further research is needed to ensure that it minimally impacts amphibian health, especially before the assay is performed on more vulnerable species.

#### Experimental controls

It is well established that appropriate controls are an essential part of scientific study. In the case of PHA studies, the approach to implementing controls has varied. Many studies evaluated PHA-associated swelling by injecting PHA into one limb, while injecting PBS into the other limb as a control (Table 1). It is possible that PHA injections can have a systemic effect, meaning that these bilateral controls are not ‘true’ controls. Other studies, primarily of anuran larvae and salamanders, made injections in the tail rather than legs. All but one of these studies (Ceccato *et al.*, 2016) added a separate control group of animals that received PBS control injections instead of PHA, a practice referred to hereafter as ‘whole animal controls’. One larval study included a third control group that received no injection at all (Murillo-Rincón *et al.*, 2017).

Nearly 20% of studies did not use controls of any kind. These studies generally cited research by Smits *et al.* (1999), showing that eliminating controls reduced variation, sampling time, handling stress and measurement variability, and that controls only served to identify two outliers out of 608 specimens tested, leading the authors to suggest it would be best to eliminate controls in these studies. However, no such analysis has ever been performed on amphibians. Furthermore, controls are necessary to account for the compression effect, discussed above in **Experimental Variables: Swelling Response** (Clulow *et al.*, 2015). While rarely acknowledged in the literature or accounted for by measurement technique or statistical analysis, the compression effect has been observed in studies in which negative swelling results were found. Gervasi and Foufopoulos (2008) found these negative differences negligible, and included them in their study; however, Ceccato *et al.* (2016) found negative swelling results in many measurements, and the choice by the authors to remove these anomalous results reduced sample size significantly. For all these reasons, steps are needed to either statistically control for compression or pursue non-compressive measurement tools (e.g. digital imaging). Until then, the use of experiment controls remains a best practice for amphibian PHA studies.

#### Histology

Histological analysis is essential to establish the mechanisms behind, and interpretation of, PHA-induced oedema (Zhang *et al.*, 2017). However, only four amphibian PHA studies to date have published histological information (Brown *et al.*, 2011, 2015; Clulow *et al.*, 2015; Josserand *et al.*, 2015). Josserand *et al.* (2015) verified results from *H. arborea* using a blood smear taken 24 hours after injection, stained using May Grünwald-Giemsa, and examined using light microscopy to identify white blood cells. Measurements included leukocyte ratios per 100 white blood cells, leukocytes per 10 000 red blood cells and the ratio of neutrophils to lymphocytes. Results showed that two injections of PHA induced higher leukocyte counts per 10 000 red blood cells, as well as higher lymphocyte ratios, than a single injection alone ([Bibr ref55][Bibr ref55]). The study also found that white blood cell counts were correlated with the size of the swelling response. The authors suggested that their data support the hypothesis that initial swelling from PHA injection represents an innate response, while the second, which is heightened by inoculation, is more adaptive.

Brown *et al.* (2011, 2015) performed histological analysis of injected tissue directly in *R. marina*. Biopsies of injected skin were taken post-injection for both PHA and PBS injection sites, fixed in phosphate-buffered formalin, embedded in paraffin, stained in haematoxylin and eosin and examined using light microscopy to identify white blood cells and generate a total cell count per set field of view. The first study (Brown *et al.*, 2011) sought to use histological analysis to assess the mechanism behind PHA swelling at varying points in the process. This analysis found that the magnitude of the swelling response at PHA injection sites was positively correlated with the average number of lymphocytes per field of view, with eosinophils, neutrophils and macrophages peaking at 12 hours post-injection, and lymphocytes peaking at 24 hours post-injection, lending support to the effectiveness of the assay to assess both innate and adaptive immune system function, with optimal testing times depending on the response of interest (Brown *et al.*, 2011). The authors noted, however, that *R. marina* did not show lymphocyte mitosis in response to PHA until 3 days after injection; therefore, the swelling response at 24 hours is largely representative of recruitment of pre-existing lymphocytes (Goldshein and Cohen, 1972; Brown *et al.*, 2011). Meanwhile, PBS injection was associated with a brief increase in non-lymphocyte white blood cells, but not a significant increase in lymphocyte recruitment (Brown *et al.*, 2011). The second study (Brown *et al.*, 2015), which took histological samples only at 72 hours post-injection, found similar results, with white blood cell counts significantly higher at PHA injection sites.

Finally, Clulow *et al*.’s (2015) study of *L. aurea* and *L. peronii* conducted histological analysis at 24 hours post-injection. Biopsies of injected skin were fixed in Bouin’s fixative, embedded in paraffin, marked with Blue Tissue Marking Dye, stained in haematoxylin and eosin and examined using light microscopy to assess inflammation and identify and count white blood cells (Clulow *et al.*, 2015). Analysis showed significant vasodilation and infiltration of leukocytes in PHA injection sites (Clulow *et al.*, 2015). Cell counts were significantly higher at PHA injection sites for lymphocytes, neutrophils and macrophages, though not for eosinophils, supporting engagement of both innate and adaptive immune responses (Clulow *et al.*, 2015). Histological verification of the response to PHA provides important insights into the nature of the immune response, which is critical for understanding the interaction between amphibian hosts and their pathogens. However, given the need to sacrifice individuals to acquire this verification, it is essential to apply them only where they are most needed. Otherwise, analysis of blood samples may be preferable.

## The Role of the PHA Assay in Assessing Amphibian Immunity

A primary objective of this review is to examine the existing body of work surrounding the PHA assay in amphibians. The assay is intended to evaluate the potential of an individual to mount a localized inflammatory reaction in response to an infection. In vertebrates, increased PHA swelling is generally regarded as an indicator of immune health (Seiter, 2009; Brown and Shine, 2014; Ceccato *et al.*, 2016; Szuroczki *et al.*, 2016, 2019). Studies have shown that the PHA test can be used to measure immunocompetence in birds (Martin *et al.*, 2006; Tella *et al.*, 2008; Salaberria *et al.*, 2013). These tests indicate that the area of swelling is associated with the infiltration and counts of certain immune cells (Martin *et al.*, 2006). Furthermore, these tests also demonstrate that the PHA assay produces a stronger response after inoculation, causing T-lymphocyte proliferation after a single dose, but even greater proliferation after a subsequent dose (Tella *et al.*, 2008). There is also a positive correlation between PHA oedema and the phagocytic activity of heterophiles and monocytes (Salaberria *et al.*, 2013). While research validating the PHA assay in amphibians is not as extensive, increased PHA oedema has been consistently associated with elevated blood leukocyte levels (Gervasi and Foufopoulos, 2008; Josserand *et al.*, 2015; Szuroczki *et al.*, 2019). Histological assessment in amphibians has found immune cell infiltration at the injection site (Brown *et al.*, 2011; Josserand *et al.*, 2015; Ceccato *et al.*, 2016), and inoculation has been shown to induce larger swelling responses on subsequent injection (Brown *et al.*, 2011).

PHA assay results have also been associated with important measures of individual or population health status. PHA results in birds have been tied to body condition, survival rates, longevity, current infection, and recruitment (Navarro *et al.*, 2003; Moreno *et al.*, 2005; Sivaraman and Kumar, 2013; Bowers *et al.*, 2014). In amphibians, higher levels of PHA swelling have been linked to protein- and anti-oxidant-rich diets during the larval stage, which authors attribute to the positive impact of these diets on immune health (Venesky *et al.*, 2012; Szuroczki *et al.*, 2016, 2019). Conversely, factors associated with reduced PHA swelling in amphibians include desiccation, density stress, captivity, and predator presence, attributes which the authors suggest would reduce immunocompetence (Gervasi and Foufopoulos, 2008; Seiter, 2009; Clulow *et al.*, 2015; Titon *et al.*, 2019).

Despite strong evidence supporting PHA as a meaningful measure of immunity, the immune system is highly complex, and different immune measures that capture diverse aspects of the immune system can yield conflicting patterns (Kennedy and Nager, 2006; Martin *et al.*, 2006). For instance, Brown and Shine (2014) found that toads that moved greater distances had diminished bacteria-killing ability (BKA) and phagocytic abilities, but increased PHA oedema. The authors suggest that this difference may be the result of a trade-off in which increased energy requirements lead to reduced investment in bacteria-killing and phagocytic abilities, while leukocyte proliferation and inflammation are increased to compensate. Similarly, in the avian PHA literature, Pigeon *et al.* (2013) found a significant negative correlation between BKA and PHA results in intensive farmland habitats. Other studies, meanwhile, found positive correlations between these same immune measures. A study of *Rhinella ornata* toads found that long-term captivity suppressed both PHA swelling and BKA (Titon *et al.*, 2019), and a study of *Passer domesticus* found that PHA and BKA results were both positively correlated with the same major histocompatibility complex genes (Lukasch *et al.*, 2017). Although these conflicting patterns are not surprising given the complexity of the immune system and the limitations of any given immune test (Kennedy and Nager, 2006; Martin *et al.*, 2006), further research investigating the relationships between external factors and different aspects of the immune response is needed to elucidate the mechanisms driving variation. In addition, performing multiple immune assays, where possible, can help to identify how specific aspects of immunity are affected by internal and external factors (Martin *et al.*, 2006).

Adding to the complexity, different diseases have varying effects on immune measures, and pathogens themselves may be affected differently by specific aspects of immunity measured (Viney *et al.*, 2005). For instance, certain pathogens or contaminants, like *Bd*, may suppress components of the host immune system (Fites *et al.*, 2014). Studies on adult amphibians have shown reduced PHA swelling in response to infections caused by *Bd* and *Echinostoma trivolvis* (Fites *et al.*, 2014; Szuroczki *et al.*, 2016; Venesky and Laskey, 2022). However, some pathogens or contaminants can indirectly upregulate certain aspects of the immune system while suppressing others. Exposure to organochlorines in amphibians, for example, has resulted in increased PHA oedema, despite a suppressed antibody response to keyhole limpet haemocyanin linked to dinitrophenyl (KLH-DNP) (Gilbertson *et al.*, 2003). The authors postulated that contaminants may stimulate certain facets of the immune system or trigger compensatory upregulation to counter reduced antibody production. This phenomenon has also been observed in birds. In a study on American kestrels, exposure to polychlorinated biphenyls (PCBs) increased PHA swelling and white blood cell counts but decreased plasma triiodothyronine levels, which have been associated with reduced antibody production and T-cell response (Smits *et al.*, 2002). The researchers hypothesized that PCB exposure may directly or indirectly affect thyroid hormones, leading to a diminished antibody response and stimulated white blood cell proliferation. However, there is a lack of laboratory studies that directly link PHA results to specific disease outcomes, and such studies would greatly facilitate the interpretation of PHA studies (Owen *et al.*, 2010). Furthermore, establishing expected swelling response in a given species would enable the detection of both unusually low and unusually high swelling responses, which could indicate immune dysregulation.

## Challenges and Recommendations

There are now several dozen amphibian PHA studies in the literature that have yielded important insights into amphibian immunity. However, a lack of validation and optimization studies, substantial variation in methodology and reporting, and limited taxonomic and geographic diversity still limit its application. Here, we discuss areas where improvement is needed based on this review and make recommendations for addressing these areas with future research.

Challenge 1: **Challenge 1: Lack of validation and optimization reduces consistency and utility of results**

**Recommendation**: *Validations should be performed, and protocols optimized for all unvalidated study species, methodologies, and control schemas, including more substantial histological study.*

Validations are used to confirm the efficacy of the PHA assay for a given species or life stage. This typically involves optimizing technical variables such as measurement timing, injection concentration, and injection site, to effectively measure a swelling response to PHA. Therefore, the terms ‘validation’ and ‘optimization’ are often used interchangeably in the literature. Several validation studies have evaluated swelling measurement timing, which is essential for establishing a peak swelling response (Table 1). Since peak swelling varies from species to species, or even potentially among individuals, sexes, and life stages, this validation step increases confidence in results for a given species. Most validation studies have used the ‘24-hour regime’ using triplicate measurements, while one study used an ‘every 4-hour regime’ using single measurements. More frequent measurements are better for determining the timing of peak swelling, while recording multiple measurements increases confidence in results. However, increasing measurements also increases handling stress, risk of injury, and research effort. Ideally, the best approach is one that is more accurate and precise, and less invasive and potentially harmful. In this vein, researchers should consider working to develop digital imaging measurement techniques that require minimal handling stress and enable more accurate measurements without the risk of tissue compression. Until such a methodology is fully developed, researchers should perform optimizations on any newly tested species by measuring swelling every 4–6 hours for the first 12 hours after injection, and then at least every 12–24 hours thereafter, with preferably two or more replicates per time point. Since the ‘24-hour regime’ is the most used, and involves measurements in triplicate, it can be an effective way to keep future studies consistent. Ultimately, the establishment of a consistent measurement time, such as 24 hours used in the standard avian protocol, would be beneficial. However, such a protocol could do more harm than good if the measurement time fails to capture significant immunological data for some species, making validation of such a universal measurement time essential.

Given that the majority of PHA studies in amphibians have used control limbs rather than whole animal controls, and some studies do not use controls at all, there is a need to delineate which types of controls are/are not needed under different scenarios. While studies in birds have suggested that PHA controls are not necessarily beneficial, the same conclusion has not been adequately drawn for amphibians. Demonstrating that controls are unnecessary for amphibians under certain conditions would lend increased flexibility to field studies. In addition, salamanders and larval amphibians typically require whole animal controls because their limbs are too small or absent to receive concurrent control injections, which greatly increases the sample size. Some have argued that controls are essential in amphibian PHA studies to account for the compression effect (**Experimental Variables: Swelling Response**). However, future improvements in digital imaging-based measurements of oedema could alleviate this concern. Until such time, true whole animal controls are still generally recommended, and at least using control limbs in post-metamorphic anurans remains best practice.

In addition to general validation and optimization, more thorough histological investigation would improve our understanding of the specific lymphocytes accumulating in tissues surrounding the injection site. Although histological analyses have been performed in previous PHA studies in amphibians, there are still gaps to be addressed by future work. First, there has been no hour-to-hour analysis of cell infiltration into tissues. Second, B- and T-cell counts have not been differentiated in prior studies. Third, there has been no detailed histological comparison of immune responses between species. Finally, the influence of inoculation on histology has not been evaluated. All these comparisons would better indicate the nature and progression of the immune response to PHA and would help establish the extent to which swelling represents innate immunity, adaptive immunity or both at a given point in time. This would greatly improve the applicability of the PHA assay for predicting how a given population or species may respond to infectious diseases. The primary drawback of histological analysis is the need to sacrifice specimens; therefore, we recommend that such validations of swelling response be initially conducted at broader taxonomic levels , reserving histological analysis for detailed, species-by-species validation.

Challenge 2: **Challenge 2: Inconsistent methodologies among studies reduces utility and may cause unnecessary harm**

**Recommendation**: *Consistent, conservative methodologies are needed for measurement timing, injection site, and PHA dose.*

Certain methodological aspects of PHA studies in amphibians are highly inconsistent across studies. As discussed above, many studies have attempted to measure peak swelling, often at a ‘default’ time such as 24 hours, while others have measured swelling at earlier time points. Injection site has also varied substantially (Fig. 3). Toe and toe web injections have been found to be effective for larger adult frogs, but other body forms and sizes require alternative injection sites. PHA dose, however, is the area that would most benefit from greater consistency, varying dramatically from study to study, from 0.01 to 20 mg. While protocols exist for optimizing peak swelling timing for untested species, there is no protocol for determining PHA dose or injection site. Injection of 0.5 mg of PHA seems to be effective even for larger species, while 0.2 mg was most used, and 0.1 mg was successfully used in specimens as large as *R. marina*. Subcutaneous toe or toe web injections have been widely used in larger species, while smaller species tend to find greater success with injections further up the leg. Meanwhile, tail injections were used for salamanders and larval amphibians. Therefore, studies should consider establishing a swelling response with injections of 0.5 mg PHA either using the toe or toe web for large species, higher on the leg for smaller species, and on the tail for salamanders and larval amphibians. Only when a significant swelling response is not found using these methods do we recommend using higher concentrations of PHA. Although some research found greater success with intramuscular injections, given that there is histological evidence that PHA injections may cause muscle damage in some specimens, studies with smaller specimens should begin with subcutaneous injections on the leg, and only if unsuccessful turn to intramuscular injections. As in avian studies where the PHA protocols have been standardized, establishing a consistent dose and injection site for as many amphibian species as possible would facilitate comparisons among species and studies.

Challenge 3: **Challenge 3: Lack of consistency in reporting impedes interpretation and replication**

**Recommendation**: *Injection site, morphological features and swelling response reporting should be expanded and standardized across studies.*

Reporting of injection site, swelling response, and basic information about test animals (e.g. weight and SVL) has varied widely across studies, impeding study comparability and study design development. Many studies do not provide sufficient detail of injection sites to allow for duplication. Based on the avian literature, swelling responses are not likely comparable among swelling sites, further supporting the need for standardization. To address this problem, procedures like those found in Venesky *et al.* (2012) are recommended, in which a specific measurement from a consistent reference point (e.g. tip of the tail) was used. Ideally, a similar location would be generally used across studies within the same species and metamorphic stage, and, where possible, other species with similar morphological attributes.

Swelling response reporting has also been inconsistent. To enable comparison between studies, a consistent reporting methodology, ideally coupled with a full dataset of starting and peak measurements for both PHA and PBS injection sites, should be reported. We recommend adopting the percentage methodology of Brown *et al.* (2015) and Zhang *et al.* (2017) (Equation1).

(1) $$ \small\begin{align*} 100&\times \left(\frac{PHA\ thickness\ at\ x\ hour- PHA\ thickness\ at\ 0\ hour}{PHA\ thickness\ at\ 0\ hour}\ \right)\notag \\ &- \left(\frac{Saline\ thickness\ at\ x\ hour- Saline\ thickness\ at\ 0\ hour}{Saline\ thickness\ at\ 0\ hour}\right) \end{align*}

Some validation studies are performed as a pilot, but go unreported, making their findings less accessible for future study of the species (de Assis *et al.*, 2015; Venesky and Laskey, 2022). Reporting these findings in supplemental information, as in Zhang *et al.* (2017), would facilitate future study and repeatability.

Finally, studies should provide raw data in publications, when possible, in accordance with the FAIR (findable, accessible, interoperable and reusable) principles. It is common not only in PHA studies, but also many other biological disciplines, to present summary statistics for each experimental treatment group or replicate, but this makes it difficult to compare results among studies and improve the PHA assay through meta-analysis. More thorough reporting of basic information for each individual including size (both SVL and weight) and swelling measurements as supplemental materials would greatly enhance protocols for PHA testing in amphibians. We recommend that datasets be deposited in permanent data repositories upon publication. These datasets should be easy to find and freely accessible to increase their applicability in conservation biology.

Challenge 4: **Challenge 4: Taxonomic and geographic biases reduce conservation applicability**

**Recommendation**: *An effort should be made to apply the PHA assay to under-represented taxonomic groups and geographic areas.*

Given its affordability, ease of use and relatively minimal impact on specimens, the PHA assay is uniquely suited to testing a wide array of amphibian groups worldwide. Historically, however, the assay’s application has been limited, with many taxonomic groups and geographic areas underrepresented in the literature. More studies are needed on caudates, amphibians from South America and Africa, and data-deficient families such as *Microhylidae*, *Ceratobatrachidae* and *Strabomantidae*. To begin with, studying species that are easily obtainable or have known husbandry such as *Leptopelis calcaratus* (Africa) or *Dendrobates pumilio* (South America) could be a potential next step.

Challenge 5: **Challenge 5: Further research needed to support field implementation and explore effects of PHA assay on amphibians**

**Recommendation**: *Further laboratory study is needed to establish the impact of organismal and environmental variables on PHA assay results, and the effects of the PHA assay on animal welfare, both short- and long-term. Once these gaps have been addressed, field-specific protocols can be established, and field study undertaken.*

To expand the applicability of the PHA assay, a greater understanding of the impact of organismal and environmental variables on assay results must be established. Once these variables are sufficiently understood to interpret the results of the PHA assay in the field, field-specific protocols must be established and validated. In addition, the effects of the assay itself on specimen mobility, health and stress, especially during active swelling, should be studied to ensure it does not harm animals.

The impact of organismal and environmental variables can be assessed via laboratory validation and targeted study. Once this gap has been addressed, field protocols may be established by (1) identifying and validating (via histology or similar immunological testing) the earliest possible swelling response to allow full testing within a single day (optimized in the lab), or alternatively, overnight housing in containers that generate a minimal stress response (optimized in the field), (2) creating consistent injection and swelling measurement protocols that can be replicated under field conditions and (3) identifying and, where possible, addressing environmental factors that could affect field results. In addition, effects on specimens, particularly in the first 72 hours, should be carefully evaluated. To date, no study has specifically sought to identify the effect of the PHA assay on mobility, health or stress. Future studies that directly measure mobility, stress hormones, food consumption and other measures of health and fitness, are essential to establish the field viability of this test and to assess the viability of its use in vulnerable species.

Challenge 6: **Challenge 6: Further investigate relationships between the PHA assay and disease outcomes**

**Recommendation**: *Further study should be undertaken to establish the relationships between PHA assay results and specific pathogens and contaminants across species.*

While a significant swelling response to PHA is generally associated with immunocompetence, and the lack thereof indicates immunosuppression, there are exceptions. The complexity of the innate immune response or disease conditions supports the need for further research on the relationships between disease outcomes and PHA results in a particular species. It is crucial to study both how various diseases and contaminants influence a species' PHA response, and what these PHA results can reveal about potential disease outcomes. To this end, we recommend that the following types of studies are conducted: 1) species-specific analysis to establish baseline PHA responses across a range of healthy individuals, 2) disease-focused investigations that evaluate the impact of diseases of interest on a species’ PHA response, and 3) contaminant studies that investigate the potential influence of contaminants and other environmental stressors on PHA responses. Where feasible, we also recommend that multiple measures of immunity be gathered to generate as complete a picture of immune health as possible.

## Conclusion

In the past 2 decades, a foundation has been built for applying the PHA assay to amphibian immunology and conservation. Such tools are more important than ever given the rise of multiple emerging infectious diseases in amphibians and their many interactions with global environmental change. To date, there have been 36 PHA studies on 27 amphibian species, spanning a wide variety of research goals. In this review, we have outlined a set of improvements that would enable the PHA assay to be more broadly and consistently applied across amphibian taxa. Detailed protocols and optimization are needed for all test species, including the determination of swelling measurement times, injection sites, and PHA injection concentrations, confirmation of the swelling response via histological analysis and the use of appropriate experimental controls. The results of all PHA studies, including validation studies, should have consistent and detailed reporting, including enough information to allow replication or meta-analysis. Such information includes basic morphological characteristics such as size and weight, as well as swelling response for each individual tested, and clear communication of injection site. Additional effort should be made to conduct studies with species from underrepresented taxa and geographic areas. More work is needed to develop protocols that are amenable for field use, including confirming peak swelling timing for species and evaluating animal welfare post-test. Adaptation of the PHA assay to the field will help address ecological questions that may not be easily answered with model organisms in the lab. Finally, better communication among researchers utilizing the PHA assay in amphibians will help increase the consistency and utility of studies, which could be achieved, for example, through the development of society meeting sessions or workshops or the creation of common study protocols and templates.

## Supplementary Material

Web_Material_coad090

## References

[ref1] Abu Bakar A , Bower DS, Stockwell MP, Clulow S, Clulow J, Mahony MJ (2016) Susceptibility to disease varies with ontogeny and immunocompetence in a threatened amphibian. Oecologia 181: 997–1009. 10.1007/s00442-016-3607-4. 27021312

[ref2] Al-Khalaifah H , Al-Nasser A (2020) Dietary supplementation with various fat oils affect phytohemagglutinin skin test in broiler chickens. Front Immunol 11: 1735. 10.3389/fimmu.2020.01735. 32922388 PMC7456851

[ref3] Arct A , Drobniak SM, Mellinger S, Gustafsson L, Cichoń M (2019) Parental genetic similarity and offspring performance in blue tits in relation to brood size manipulation. Ecol Evol 9: 10085–10091. 10.1002/ece3.5367. 31624539 PMC6787802

[ref4] de Assis VR , Titon SCM, Barsotti AMG, Titon B Jr, Gomes FR (2015) Effects of acute restraint stress, prolonged captivity stress and transdermal corticosterone application on immunocompetence and plasma levels of corticosterone on the Cururu toad (Rhinella icterica). PloS One 10: e0121005. 10.1371/journal.pone.0121005. 25831055 PMC4382218

[ref5] Bakewell L , Kelehear C, Graham SP (2021) Impacts of temperature on immune performance in a desert anuran (*Anaxyrus punctatus*). J Zool 315: 49–57. 10.1111/jzo.12891.

[ref6] Bienentreu J-F , Lesbarrères D (2020) Amphibian disease ecology: are we just scratching the surface? Herpetologica 76: 153–166. 10.1655/0018-0831-76.2.153.

[ref7] Bishop PJ , Angulo A, Lewis JP, Moore RD, Rabb GB, Moreno JG (2012) The amphibian extinction crisis - what will it take to put the action into the amphibian conservation action plan? SAPIENS Surv Perspect Integrating Environ Soc.

[ref8] Blaustein AR , Gervasi SS, Johnson PTJ, Hoverman JT, Belden LK, Bradley PW, Xie GY (2012) Ecophysiology meets conservation: understanding the role of disease in amphibian population declines. Philos Trans R Soc B Biol Sci 367: 1688–1707. 10.1098/rstb.2012.0011. PMC3350657 22566676

[ref9] Bolochio BE , Lescano JN, Cordier JM, Loyola R, Nori J (2020) A functional perspective for global amphibian conservation. Biol Conserv 245: 108572. 10.1016/j.biocon.2020.108572.

[ref10] Bowers EK , Hodges CJ, Forsman AM, Vogel LA, Masters BS, Johnson BGP, Johnson LS, Thompson CF, Sakaluk SK (2014) Neonatal body condition, immune responsiveness, and hematocrit predict longevity in a wild bird population. Ecology 95: 3027–3034. 10.1890/14-0418.1. 25505800 PMC4260523

[ref11] Brannelly LA , Ohmer MEB, Saenz V, Richards-Zawacki CL (2019) Effects of hydroperiod on growth, development, survival and immune defences in a temperate amphibian. Funct Ecol 33: 1952–1961. 10.1111/1365-2435.13419.

[ref12] Brown GP , Kelehear C, Shilton CM, Phillips BL, Shine R (2015) Stress and immunity at the invasion front: a comparison across cane toad (*Rhinella marina*) populations. Biol J Linn Soc 116: 748–760. 10.1111/bij.12623.

[ref13] Brown GP , Shilton CM, Shine R (2011) Measuring amphibian immunocompetence: validation of the phytohemagglutinin skin-swelling assay in the cane toad, *Rhinella marina*. Methods Ecol Evol 2: 341–348. 10.1111/j.2041-210X.2011.00090.x.

[ref14] Brown GP , Shine R (2014) Immune response varies with rate of dispersal in invasive cane toads (*Rhinella marina*). PloS One 9: e99734. 10.1371/journal.pone.0099734. 24936876 PMC4061023

[ref15] Brown SR , Flynn RW, Hoverman JT (2021) Perfluoroalkyl substances increase susceptibility of northern leopard frog tadpoles to trematode infection. Environ Toxicol Chem 40: 689–694. 10.1002/etc.4678. 31995841

[ref16] Brunner JL , Storfer A, Gray MJ, Hoverman JT (2015) Ranavirus ecology and evolution: from epidemiology to extinction. In MJ Gray, VG Chinchar, eds, Ranaviruses: Lethal Pathogens of Ectothermic Vertebrates. Springer International Publishing, Cham, pp. 71–104.

[ref17] Butler RA (2019) Total number of amphibian species, by country. Mongabay . https://rainforests.mongabay.com/03amphibian.htm last accessed 21 December 2022.

[ref18] Cardillo M (2021) Clarifying the relationship between body size and extinction risk in amphibians by complete mapping of model space. Proc R Soc B Biol Sci 288: 20203011. 10.1098/rspb.2020.3011. PMC7893221 33529561

[ref19] Carey C , Cohen N, Rollins-Smith L (1999) Amphibian declines: an immunological perspective. Dev Comp Immunol 23: 459–472. 10.1016/S0145-305X(99)00028-2. 10512457

[ref20] Cassettari BO , Madelaire CB, Gomes FR (2022) Elevated corticosterone levels are associated with increased immunocompetence in male toads, both when calling and under experimental conditions. Horm Behav 137: 105083. 10.1016/j.yhbeh.2021.105083. 34773784

[ref21] Ceccato E , Cramp RL, Seebacher F, Franklin CE (2016) Early exposure to ultraviolet-B radiation decreases immune function later in life. Conserv Physiol 4: cow037. 10.1093/conphys/cow037. 27668081 PMC5033135

[ref22] Christie AP , Amano T, Martin PA, Petrovan SO, Shackelford GE, Simmons BI, Smith RK, Williams DR, Wordley CFR, Sutherland WJ (2020) Poor availability of context-specific evidence hampers decision-making in conservation. Biol Conserv 248: 108666. 10.1016/j.biocon.2020.108666.

[ref23] Christie AP , Amano T, Martin PA, Petrovan SO, Shackelford GE, Simmons BI, Smith RK, Williams DR, Wordley CFR, Sutherland WJ (2021) The challenge of biased evidence in conservation. Conserv Biol 35: 249–262. 10.1111/cobi.13577. 32583521

[ref24] Christin M-S , Gendron AD, Brousseau P, Ménard L, Marcogliese DJ, Cyr D, Ruby S, Fournier M (2003) Effects of agricultural pesticides on the immune system of Rana pipiens and on its resistance to parasitic infection. Environ Toxicol Chem 22: 1127–1133. 10.1002/etc.5620220522. 12729224

[ref25] Clulow S , Harris M, Mahony MJ (2015) Optimization, validation and efficacy of the phytohaemagglutinin inflammation assay for use in ecoimmunological studies of amphibians. Conserv Physiol 3: cov042. 10.1093/conphys/cov042. 27293727 PMC4778488

[ref26] Collins DS , Sánchez-Félix M, Badkar AV, Mrsny R (2020) Accelerating the development of novel technologies and tools for the subcutaneous delivery of biotherapeutics. J Control Release 321: 475–482. 10.1016/j.jconrel.2020.02.036. 32105759

[ref27] Collins JP (2010) Amphibian decline and extinction: what we know and what we need to learn. Dis Aquat Organ 92: 93–99. 10.3354/dao02307. 21268970

[ref28] Collins JP , Halliday T (2005) Forecasting changes in amphibian biodiversity: aiming at a moving target. Philos Trans R Soc B Biol Sci 360: 309–314. 10.1098/rstb.2004.1588. PMC1569460 15856554

[ref29] Cornuau JH , Schmeller DS, Pigeault R, Loyau A (2014) Resistance of morphological and behavioral sexual traits of the palmate newt (*Lissotriton helveticus*) to bacterial lipopolysaccharide treatment. Amphib-Reptil 35: 63–71.

[ref30] Daszak P , Cunningham AA, Hyatt AD (2003) Infectious disease and amphibian population declines. Diversity and Distributions 9: 141–150. 10.1046/j.1472-4642.2003.00016.x.

[ref31] Demas GE , Zysling DA, Beechler BR, Muehlenbein MP, French SS (2011) Beyond phytohaemagglutinin: assessing vertebrate immune function across ecological contexts: assessing vertebrate immune function across ecological contexts. J Anim Ecol 80: 710–730. 10.1111/j.1365-2656.2011.01813.x. 21401591

[ref32] Dempsey PW , Vaidya SA, Cheng G (2003) The art of war: innate and adaptive immune responses. Cell Mol Life Sci 60: 2604–2621. 10.1007/s00018-003-3180-y. 14685686 PMC11138847

[ref33] Desprat JL , Lengagne T, Dumet A, Desouhant E, Mondy N (2015) Immunocompetence handicap hypothesis in tree frog: trade-off between sexual signals and immunity? Behav Ecol 26: 1138–1146. 10.1093/beheco/arv057.

[ref34] Desprat JL , Lengagne T, Mondy N (2017) Immune challenges and visual signalling in tree frogs. Sci Nat 104: 21. 10.1007/s00114-017-1436-x. 28271177

[ref35] Fernández-Benéitez MJ , Ortiz-Santaliestra ME, Lizana M, Diéguez-Uribeondo J (2008) *Saprolegnia diclina*: another species responsible for the emergent disease ‘Saprolegnia infections’ in amphibians. FEMS Microbiol Lett 279: 23–29. 10.1111/j.1574-6968.2007.01002.x. 18177304

[ref36] Finger JW , Thomson PC, Adams AL, Benedict S, Moran C, Isberg SR (2015) Reference levels for corticosterone and immune function in farmed saltwater crocodiles (*Crocodylus porosus*) hatchlings using current code of practice guidelines. Gen Comp Endocrinol 212: 63–72. 10.1016/j.ygcen.2015.01.023. 25644211

[ref37] Fites JS , Reinert LK, Chappell TM, Rollins-Smith LA (2014) Inhibition of local immune responses by the frog-killing fungus *Batrachochytrium dendrobatidis*. Infect Immun 82: 4698–4706. 10.1128/IAI.02231-14. 25156734 PMC4249309

[ref38] Flajnik MF , Hsu E, Kaufman JF, Pasquier LD (1987) Changes in the immune system during metamorphosis of *Xenopus*. Immunol Today 8: 58–64. 10.1016/0167-5699(87)90240-4. 25291685

[ref39] Ford KL , Albert JS, Summers AP, Hedrick BP, Schachner ER, Jones AS, Evans K, Chakrabarty P (2023) A new era of morphological investigations: reviewing methods for comparative anatomical studies. Integrative Organismal Biology 5: obad008. 10.1093/iob/obad008. 37035037 PMC10081917

[ref40] Forson DD , Storfer A (2006) Atrazine increases Ranavirus susceptibility in the Tiger salamander, *Ambystoma Tigrinum*. Ecol Appl 16: 2325–2332. 10.1890/1051-0761(2006)016[2325:AIRSIT]2.0.CO;2. 17205907

[ref41] French SS , McLemore R, Vernon B, Johnston GIH, Moore MC (2007) Corticosterone modulation of reproductive and immune systems trade-offs in female tree lizards: long-term corticosterone manipulations via injectable gelling material. J Exp Biol 210: 2859–2865. 10.1242/jeb.005348. 17690234

[ref42] Gervasi SS , Foufopoulos J (2008) Costs of plasticity: responses to desiccation decrease post-metamorphic immune function in a pond-breeding amphibian. Funct Ecol 22: 100–108. 10.1111/j.1365-2435.2007.01340.x.

[ref43] Gilbertson M-K , Haffner GD, Drouillard KG, Albert A, Dixon B (2003) Immunosuppression in the northern leopard frog (*Rana pipiens*) induced by pesticide exposure. Environ Toxicol Chem 22: 101–110. 10.1002/etc.5620220113. 12503752

[ref44] Goetz SM , Romagosa CM, Appel AG, Guyer C, Mendonça MT (2017) Reduced innate immunity of Cuban treefrogs at leading edge of range expansion. J Exp Zool Part Ecol Integr Physiol 327: 592–599. 10.1002/jez.2146. 29527833

[ref45] Goldshein SJ , Cohen N (1972) Phylogeny of immunocompetent cells: I. In vitro blastogenesis and mitosis of toad (*Bufo Marinus*) splenic lymphocytes in response to phytohemagglutinin and in mixed lymphocyte cultures1. J Immunol 108: 1025–1033. 10.4049/jimmunol.108.4.1025. 5023165

[ref46] Grasman KA (2010) In vivo functional tests for assessing immunotoxicity in birds. In RR Dietert, ed, Immunotoxicity Testing: Methods and Protocols. Humana Press, Totowa, NJ, pp. 387–398 10.1007/978-1-60761-401-2_25 19967526

[ref47] Grasman KA , Fox GA, Scanlon PF, Ludwig JP (1996) Organochlorine-associated immunosuppression in prefledgling Caspian terns and herring gulls from the Great Lakes: an ecoepidemiological study. Environ Health Perspect 104 Suppl 4: 829–842. 8880006 10.1289/ehp.96104s4829 PMC1469670

[ref48] Greer AL , Collins JP (2008) Habitat fragmentation as a result of biotic and abiotic factors controls pathogen transmission throughout a host population. J Anim Ecol 77: 364–369. 10.1111/j.1365-2656.2007.01330.x. 18005032

[ref49] Grogan LF , Robert J, Berger L, Skerratt LF, Scheele BC, Castley JG, Newell DA, McCallum HI (2018) Review of the amphibian immune response to chytridiomycosis, and future directions. Front Immunol 9: 2536. 10.3389/fimmu.2018.02536. 30473694 PMC6237969

[ref50] Haislip NA , Gray MJ, Hoverman JT, Miller DL (2011) Development and disease: how susceptibility to an emerging pathogen changes through anuran development. PloS One 6: e22307. 10.1371/journal.pone.0022307. 21799820 PMC3142128

[ref144] Hasselquist D (2007) Comparative immunoecology in birds: hypotheses and tests. J Ornithol 148: 571–582.

[ref51] Hayes TB , Falso P, Gallipeau S, Stice M (2010) The cause of global amphibian declines: a developmental endocrinologist’s perspective. J Exp Biol 213: 921–933. 10.1242/jeb.040865. 20190117 PMC2829317

[ref52] Heatley JJ , Johnson M (2009) Clinical technique: amphibian hematology: a practitioner’s guide. J Exot Pet Med 18: 14–19. 10.1053/j.jepm.2008.10.004.

[ref53] Hernández-Arciga U , Herrera M. LG, Ibáñez-Contreras A, Miranda-Labra RU, Flores-Martínez JJ, Königsberg M (2018) Baseline and post-stress seasonal changes in immunocompetence and redox state maintenance in the fishing bat *Myotis vivesi*. PloS One 13: e0190047. 10.1371/journal.pone.0190047. 29293551 PMC5749750

[ref54] Iglesias-Carrasco M , Head ML, Cabido C (2018) Effect of an immune challenge on the anti-predator response of the green Iberian frog (*Pelophylax perezi*): the influence of urban habitats. Biol J Linn Soc 124: 447–455. 10.1093/biolinnean/bly051.

[ref55] Josserand R , Troïanowski M, Grolet O, Desprat JL, Lengagne T, Mondy N (2015) A phytohaemagglutinin challenge test to assess immune responsiveness of European tree frog *Hyla arborea*. Amphib-Reptil 36: 111–118.

[ref143] Kean RP , Lamont SJ (1994) Effect of Injection Site on Cutaneous Basophil Hypersensitivity Response to Phytohemagglutinin. Poult. Sci. 73: 1763–1765. 7862616 10.3382/ps.0731763

[ref56] Kennedy MW , Nager RG (2006) The perils and prospects of using phytohaemagglutinin in evolutionary ecology. Trends Ecol Evol 21: 653–655. 10.1016/j.tree.2006.09.017. 17028055

[ref57] Kerimov AB , Ilyina TA, Ivankina EV, Bushuev AV, Sokolova OV, Rogovin KA (2018) Melanin-based coloration and immunity in polymorphic population of pied flycatcher, *Ficedula hypoleuca*. Evol Ecol 32: 89–111. 10.1007/s10682-017-9926-z.

[ref58] Knapp RA , Fellers GM, Kleeman PM, Miller DAW, Vredenburg VT, Rosenblum EB, Briggs CJ (2016) Large-scale recovery of an endangered amphibian despite ongoing exposure to multiple stressors. Proc Natl Acad Sci 113: 11889–11894. 10.1073/pnas.1600983113. 27698128 PMC5081604

[ref59] LaMonica LE , Fox RJ, Donelson JM (2021) Thermal sensitivity of juvenile rabbitfishes *Siganus doliatus* and *S. Lineatus (Siganidae)*: a key role for habitat? Coral Reefs 40: 1307–1320. 10.1007/s00338-021-02146-2.

[ref60] Langerveld AJ , Celestine R, Zaya R, Mihalko D, Ide CF (2009) Chronic exposure to high levels of atrazine alters expression of genes that regulate immune and growth-related functions in developing Xenopus laevis tadpoles. Environ Res 109: 379–389. 10.1016/j.envres.2009.01.006. 19272595

[ref61] Levy DL , Heald R (2016) Biological scaling problems and solutions in amphibians. Cold Spring Harb Perspect Biol 8: a019166. 10.1101/cshperspect.a019166. PMC4691792 26261280

[ref62] Licht P , McCreery BR, Barnes R, Pang R (1983) Seasonal and stress related changes in plasma gonadotropins, sex steroids, and corticosterone in the bullfrog, *Rana catesbeiana*. Gen Comp Endocrinol 50: 124–145. 10.1016/0016-6480(83)90249-6. 6406295

[ref63] Lips KR (2016) Overview of chytrid emergence and impacts on amphibians. Philos Trans R Soc B Biol Sci 371: 20150465. 10.1098/rstb.2015.0465. PMC5095542 28080989

[ref64] Lopez-Antia A , Ortiz-Santaliestra ME, Camarero PR, Mougeot F, Mateo R (2015) Assessing the risk of fipronil-treated seed ingestion and associated adverse effects in the red-legged partridge. Environ Sci Technol 49: 13649–13657. 10.1021/acs.est.5b03822. 26448319

[ref65] Lukasch B , Westerdahl H, Strandh M, Winkler H, Moodley Y, Knauer F, Hoi H (2017) Genes of the major histocompatibility complex highlight interactions of the innate and adaptive immune system. PeerJ 5: e3679. 10.7717/peerj.3679. 28875066 PMC5581531

[ref66] Madelaire CB , Cassettari BO, Gomes FR (2019) Immunomodulation by testosterone and corticosterone in toads: experimental evidences from transdermal application. Gen Comp Endocrinol 273: 227–235. 10.1016/j.ygcen.2018.09.005. 30195026

[ref67] Madelaire CB , Sokolova I, Gomes FR (2017) Seasonal patterns of variation in steroid plasma levels and immune parameters in anurans from Brazilian semiarid area. Physiol Biochem Zool 90: 415–433. 10.1086/691202. 28398155

[ref68] Mangoni ML (2006) Temporins, anti-infective peptides with expanding properties. Cell Mol Life Sci CMLS 63: 1060–1069. 10.1007/s00018-005-5536-y. 16572270 PMC11136197

[ref69] Martin LB , Han P, Lewittes J, Kuhlman JR, Klasing KC, Wikelski M (2006) Phytohemagglutinin-induced skin swelling in birds: histological support for a classic immunoecological technique. Funct Ecol 20: 290–299. 10.1111/j.1365-2435.2006.01094.x.

[ref70] Martínez J , Merino S, Badás EP, Almazán L, Moksnes A, Barbosa A (2018) Hemoparasites and immunological parameters in snow bunting (*Plectrophenax nivalis*) nestlings. Polar Biol 41: 1855–1866. 10.1007/s00300-018-2327-0.

[ref71] Mauel MJ , Miller DL, Frazier KS, Hines ME (2002) Bacterial pathogens isolated from cultured bullfrogs (Rana castesbeiana). J Vet Diagn Invest 14: 431–433. 10.1177/104063870201400515. 12296400

[ref72] Merino S , Martinez J, Møller AP, Sanabria L, de Lope F, Perez J, Rodriguez-Caabeiro F (1999) Phytohaemagglutinin injection assay and physiological stress in nestling house martins. Anim Behav 58: 219–222. 10.1006/anbe.1999.1127. 10413560

[ref73] Merlo JL , Cutrera AP, Kittlein MJ, Zenuto RR (2018) Individual condition and inflammatory response to PHA in the subterranean rodent *Ctenomys talarum* (Talas tuco-tuco): a multivariate approach. Mamm Biol 90: 47–54. 10.1016/j.mambio.2018.02.007.

[ref74] Miller D , Gray M, Storfer A (2011) Ecopathology of ranaviruses infecting amphibians. Viruses 3: 2351–2373. 10.3390/v3112351. 22163349 PMC3230856

[ref75] Minias P , Gach K, Włodarczyk R, Janiszewski T (2019) Colony size affects nestling immune function: a cross-fostering experiment in a colonial waterbird. Oecologia 190: 333–341. 10.1007/s00442-019-04402-3. 31004188 PMC6571091

[ref76] Miyazawa R , Iijima Y, Nakanishi T (2022) Induction of both local and systemic immunity by in vivo injection of PHA into ginbuna carp fin. Dev Comp Immunol 129: 104329. 10.1016/j.dci.2021.104329. 34919981

[ref77] Moher D , Liberati A, Tetzlaff J, Altman DG, Prisma Group (2009) Preferred reporting items for systematic reviews and meta-analyses: the PRISMA statement. PLoS Med 6: e1000097. 10.1371/journal.pmed.1000097. 19621072 PMC2707599

[ref78] Møller AP , Cassey P (2004) On the relationship between T-cell mediated immunity in bird species and the establishment success of introduced populations. J Anim Ecol 73: 1035–1042. 10.1111/j.0021-8790.2004.00879.x.

[ref79] Møller AP , Saino N (2004) Immune response and survival. Oikos 104: 299–304. 10.1111/j.0030-1299.2004.12844.x.

[ref80] Moreno J , Merino S, Sanz J, Arriero E, Morales J, Tomás G (2005) Nestling cell-mediated immune response, body mass and hatching date as predictors of local recruitment in the pied flycatcher *Ficedula hypoleuca*. J Avian Biol 36: 251–260. 10.1111/j.0908-8857.2005.03413.x.

[ref81] Mougeot F , Irvine JR, Seivwright L, Redpath SM, Piertney S (2004) Testosterone, immunocompetence, and honest sexual signaling in male red grouse. Behav Ecol 15: 930–937. 10.1093/beheco/arh087.

[ref82] Murillo-Rincón AP , Laurila A, Orizaola G (2017) Compensating for delayed hatching reduces offspring immune response and increases life-history costs. Oikos 126: 565–571. 10.1111/oik.04014.

[ref83] Nasri I , Hammouda A, Belliure J, Selmi S (2020) Decreased cell-mediated immune response in Bosk’s fringe-toed lizards (*Acanthodactylus boskianus*) inhabiting an industrialized area in southern Tunisia. Bull Environ Contam Toxicol 105: 393–396. 10.1007/s00128-020-02943-5. 32699910

[ref84] Navarro C , Marzal A, De Lope F, Møller AP (2003) Dynamics of an immune response in house sparrows Passer domesticus in relation to time of day, body condition and blood parasite infection. Oikos 101: 291–298. 10.1034/j.1600-0706.2003.11663.x.

[ref85] North AC , Hodgson DJ, Price SJ, Griffiths AGF (2015) Anthropogenic and ecological drivers of amphibian disease (Ranavirosis). PloS One 10: e0127037. 10.1371/journal.pone.0127037. 26039741 PMC4454639

[ref86] Nunn CL , Lindenfors P, Pursall ER, Rolff J (2009) On sexual dimorphism in immune function. Philos Trans R Soc B Biol Sci 364: 61–69. 10.1098/rstb.2008.0148. PMC2666693 18926977

[ref87] O’Connor CM , Reddon AR, Marsh-Rollo SE, Hellmann JK, Ligocki IY, Hamilton IM, Balshine S (2014) A comparative study of an innate immune response in Lamprologine cichlid fishes. Naturwissenschaften 101: 839–849. 10.1007/s00114-014-1225-8. 25135814

[ref88] Obomsawin AP , Mastromonaco GF, Leonard ML (2021) Chronic noise exposure has context-dependent effects on stress physiology in nestling tree swallows (*Tachycineta bicolor*). Gen Comp Endocrinol 311: 113834. 10.1016/j.ygcen.2021.113834. 34181934

[ref89] Ohmer MEB , Alton LA, Cramp RL (2020) Physiology provides a window into how the multi-stressor environment contributes to amphibian declines. In CL Madliger, CE Franklin, OP Love, SJ Cooke, eds, Conservation Physiology: Applications for Wildlife Conservation and Management. Oxford University Press, pp. 165–182.

[ref90] Oliveira BF , São-Pedro VA, Santos-Barrera G, Penone C, Costa GC (2017) AmphiBIO, a global database for amphibian ecological traits. Sci Data 4: 170123. 10.1038/sdata.2017.123. 28872632 PMC5584397

[ref91] Owen JP , Nelson AC, Clayton DH (2010) Ecological immunology of bird-ectoparasite systems. Trends Parasitol 26: 530–539. 10.1016/j.pt.2010.06.005. 20599426

[ref92] Page MJ , McKenzie JE, Bossuyt PM, Boutron I, Hoffmann TC, Mulrow CD, Shamseer L, Tetzlaff JM, Akl EA, Brennan SE et al. (2021) The PRISMA 2020 statement: an updated guideline for reporting systematic reviews. Syst Rev 10: 89. 10.1186/s13643-021-01626-4. 33781348 PMC8008539

[ref93] Pigeon G , Bélisle M, Garant D, Cohen AA, Pelletier F (2013) Ecological immunology in a fluctuating environment: an integrative analysis of tree swallow nestling immune defense. Ecol Evol 3: 1091–1103. 10.1002/ece3.504. 23610646 PMC3631416

[ref94] Plasman M , Sandoval-Zapotitla E, Torres R (2019) Immune response declines with age in a wild lizard. Biol J Linn Soc 128: 936–943. 10.1093/biolinnean/blz150.

[ref95] Priyadarshani S , Madhushani WAN, Jayawardena UA, Wickramasinghe DD, Udagama PV (2015) Heavy metal mediated immunomodulation of the Indian green frog, *Euphlyctis hexadactylus* (Anura:Ranidae) in urban wetlands. Ecotoxicol Environ Saf 116: 40–49. 10.1016/j.ecoenv.2015.02.037. 25754457

[ref96] Pusch EA , Navara KJ (2018) Behavioral phenotype relates to physiological differences in immunological and stress responsiveness in reactive and proactive birds. Gen Comp Endocrinol 261: 81–88. 10.1016/j.ygcen.2018.01.027. 29410134

[ref97] Rodriguez KM , Voyles J (2020) The amphibian complement system and chytridiomycosis. J Exp Zool Part Ecol Integr Physiol 333: 706–719. 10.1002/jez.2419. PMC7821119 33052039

[ref142] Rohr JR , Raffel TR (2010) Linking global climate and temperature variability to widespread amphibian declines puta tively caused by disease. Proceedings of the National Academy of Sciences 107: 8269–8274. 10.1073/pnas.0912883107 PMC2889522 20404180

[ref98] Rohr JR , Raffel TR, Halstead NT, McMahon TA, Johnson SA, Boughton RK, Martin LB (2013) Early-life exposure to a herbicide has enduring effects on pathogen-induced mortality. Proc Biol Sci 280: 20131502. 10.1098/rspb.2013.1502. 24266041 PMC3813324

[ref99] Rohr JR , Schotthoefer AM, Raffel TR, Carrick HJ, Halstead N, Hoverman JT, Johnson CM, Johnson LB, Lieske C, Piwoni MD et al. (2008) Agrochemicals increase trematode infections in a declining amphibian species. Nature 455: 1235–1239. 10.1038/nature07281. 18972018

[ref100] Rollins-Smith LA (1998) Metamorphosis and the amphibian immune system. Immunol Rev 166: 221–230. 10.1111/j.1600-065X.1998.tb01265.x. 9914915

[ref101] Rollins-Smith LA (2017) Amphibian immunity–stress, disease, and climate change. Dev Comp Immunol 66: 111–119. 10.1016/j.dci.2016.07.002. 27387153

[ref102] Rollins-Smith LA , Woodhams DC (2011) Amphibian immunity. In G Demas, R Nelson, eds, Ecoimmunology. Oxford University Press, Incorporated, Cary UNITED STATES.

[ref103] Saad AH (1992) Zoological science. Zoolog Sci 9: 1081–1085.

[ref104] Salaberria C , Muriel J, de Luna M, Gil D, Puerta M (2013) The PHA test as an indicator of phagocytic activity in a passerine bird. PloS One 8: e84108. 10.1371/journal.pone.0084108. 24391896 PMC3877195

[ref105] Sarkis-Onofre R , Catalá-López F, Aromataris E, Lockwood C (2021) How to properly use the PRISMA statement. Syst Rev 10: 117. 10.1186/s13643-021-01671-z. 33875004 PMC8056687

[ref106] Scheele BC , Pasmans F, Skerratt LF, Berger L, Martel A, Beukema W, Acevedo AA, Burrowes PA, Carvalho T, Catenazzi A et al. (2019) Amphibian fungal panzootic causes catastrophic and ongoing loss of biodiversity. Science 363: 1459–1463. 10.1126/science.aav0379. 30923224

[ref107] Seiter SA (2009) Predator Presence Suppresses Immune Function in Larval Amphibians, Master’s thesis

[ref108] Shine R (1979) Sexual selection and sexual dimorphism in the Amphibia. Copeia 1979: 297–306. 10.2307/1443418.

[ref109] Sivaraman GK , Kumar S (2013) Immunocompetence index selection of broiler chicken lines for disease resistance and their impact on survival rate. Vet World 6: 628–631. 10.14202/vetworld.2013.628-631.

[ref110] Skerratt LF , Berger L, Speare R, Cashins S, McDonald KR, Phillott AD, Hines HB, Kenyon N (2007) Spread of chytridiomycosis has caused the rapid global decline and extinction of frogs. Ecohealth 4: 125. 10.1007/s10393-007-0093-5.

[ref111] Smits JE , Bortolotti GR, Tella JL (1999) Simplifying the phytohaemagglutinin skin-testing technique in studies of avian immunocompetence. Funct Ecol 13: 567–572. 10.1046/j.1365-2435.1999.00338.x.

[ref112] Smits JE , Fernie KJ, Bortolotti GR, Marchant TA (2002) Thyroid hormone suppression and cell-mediated immunomodulation in American kestrels (Falco sparverius) exposed to PCBs. Arch Environ Contam Toxicol 43: 338–344. 10.1007/s00244-002-1200-9. 12202931

[ref113] Smits JE , Williams TD (1999) Validation of immunotoxicology techniques in passerine chicks exposed to oil sands tailings water. Ecotoxicol Environ Saf 44: 105–112. 10.1006/eesa.1999.1806. 10499996

[ref114] Stienbarger CD , Joseph J, Athey SN, Monteleone B, Andrady AL, Watanabe WO, Seaton P, Taylor AR, Brander SM (2021) Direct ingestion, trophic transfer, and physiological effects of microplastics in the early life stages of *Centropristis striata*, a commercially and recreationally valuable fishery species. Environ Pollut 285: 117653. 10.1016/j.envpol.2021.117653. 34380229

[ref115] Szuroczki D , Koprivnikar J, Baker RL (2016) Dietary antioxidants enhance immunocompetence in larval amphibians. Comp Biochem Physiol A Mol Integr Physiol 201: 182–188. 10.1016/j.cbpa.2016.07.014. 27475300

[ref116] Szuroczki D , Koprivnikar J, Baker RL (2019) Effects of dietary antioxidants and environmental stressors on immune function and condition in *Lithobates (Rana) sylvaticus*. Comp Biochem Physiol A Mol Integr Physiol 229: 25–32. 10.1016/j.cbpa.2018.11.017. 30502473

[ref117] Taneja V (2018) Sex hormones determine immune response. Front Immunol 9: 1931. 10.3389/fimmu.2018.01931. 30210492 PMC6119719

[ref118] Tella JL , Lemus JA, Carrete M, Blanco G (2008) The PHA test reflects acquired T-cell mediated immunocompetence in birds. PloS One 3: e3295. 10.1371/journal.pone.0003295. 18820730 PMC2546448

[ref119] Thomas JR , Woodley SK (2017) Testing the immunocompetence handicap hypothesis: testosterone manipulation does not affect wound healing in male salamanders. Gen Comp Endocrinol 247: 8–15. 10.1016/j.ygcen.2017.03.014. 28343933

[ref120] Titon SCM , de Assis VR, Titon B, Barsotti AMG, Flanagan SP, Gomes FR (2016) Calling rate, corticosterone plasma levels and immunocompetence of *Hypsiboas albopunctatus*. Comp Biochem Physiol A Mol Integr Physiol 201: 53–60. 10.1016/j.cbpa.2016.06.023. 27364933

[ref121] Titon SCM , Titon B, Barsotti AMG, Gomes FR, Assis VR (2019) Time-related immunomodulation by stressors and corticosterone transdermal application in toads. PloS One 14: e0222856. 10.1371/journal.pone.0222856. 31539413 PMC6754171

[ref122] Titon SCM , Titon Junior B, Gomes FR, Assis VR (2021) Short-term stressors and corticosterone effects on immunity in male toads (*Rhinella icterica*): a neuroimmune-endocrine approach. Brain Behav Immun - Health 13: 100230. 10.1016/j.bbih.2021.100230. 34589745 PMC8474493

[ref123] Troïanowski M , Mondy N, Dumet A, Arcanjo C, Lengagne T (2017) Effects of traffic noise on tree frog stress levels, immunity, and color signaling. Conserv Biol 31: 1132–1140. 10.1111/cobi.12893. 28074559

[ref124] Vasconcelos-Teixeira R , Titon SCM, Titon B, Pompêo MLM, Gomes FR, Assis VR (2022) Stress response, immunity, and organ mass in toads (*Rhinella diptycha*) living in metal-contaminated areas. Biol Trace Elem Res 200: 800–811. 10.1007/s12011-021-02699-x. 33840055

[ref125] Venesky MD , Laskey CA (2022) Infection with *Batrachochytrium dendrobatidis* reduces salamander capacity to mount a cell-mediated immune response. J Exp Zool Part Ecol Integr Physiol 337: 273–281. 10.1002/jez.2497. 34102032

[ref126] Venesky MD , Wilcoxen TE, Rensel MA, Rollins-Smith L, Kerby JL, Parris MJ (2012) Dietary protein restriction impairs growth, immunity, and disease resistance in southern leopard frog tadpoles. Oecologia 169: 23–31. 10.1007/s00442-011-2171-1. 22038058

[ref127] Viney ME , Riley EM, Buchanan KL (2005) Optimal immune responses: immunocompetence revisited. Trends Ecol Evol 20: 665–669. 10.1016/j.tree.2005.10.003. 16701455

[ref128] Vinkler M , Bainová H, Albrecht T (2010) Functional analysis of the skin-swelling response to phytohaemagglutinin. Funct Ecol 24: 1081–1086. 10.1111/j.1365-2435.2010.01711.x.

[ref129] Vitousek MN , Taff CC, Hallinger KK, Zimmer C, Winkler DW (2018) Hormones and fitness: evidence for trade-offs in glucocorticoid regulation across contexts. Front Ecol Evol 6. 10.3389/fevo.2018.00042.

[ref130] Vredenburg VT , Knapp RA, Tunstall TS, Briggs CJ (2010) Dynamics of an emerging disease drive large-scale amphibian population extinctions. Proc Natl Acad Sci U S A 107: 9689–9694. 10.1073/pnas.0914111107. 20457913 PMC2906868

[ref131] Wang X , Zhao Z-J, Cao Y, Cui J, Tang Y, Chen J (2019) Condition dependence of advertisement calls in male African clawed frogs. J Ethol 37: 75–81. 10.1007/s10164-018-0570-z.

[ref132] Wiley JE , Meisner LF (1984) Synergistic effect of TPA and T-cell mitogens in nonmammalian vertebrates. In Vitro 20: 932–936. 10.1007/BF02619666. 6335701

[ref133] Womack M , Steigerwald E, Blackburn D, Cannatella D, Catenazzi A, Che J, Koo M, McGuire J, Ron S, Spencer C et al. (2021) State of Amphibia 2020: Five Years of Amphibian Research, Diversity and Resources

[ref134] Woodhams DC , Rollins-Smith LA, Carey C, Reinert L, Tyler MJ, Alford RA (2006) Population trends associated with skin peptide defenses against chytridiomycosis in Australian frogs. Oecologia 146: 531–540. 10.1007/s00442-005-0228-8. 16205955

[ref135] Young BE , Lips KR, Reaser JK, Ibáñez R, Salas AW, Cedeño JR, Coloma LA, Ron S, La Marca E, Meyer JR et al. (2001) Population declines and priorities for amphibian conservation in Latin America. Conserv Biol 15: 1213–1223. 10.1111/j.1523-1739.2001.00218.x.

[ref136] Young S , Whitehorn P, Berger L, Skerratt LF, Speare R, Garland S, Webb R (2014) Defects in host immune function in tree frogs with chronic chytridiomycosis. PloS One 9: e107284. 10.1371/journal.pone.0107284. 25211333 PMC4161418

[ref137] Zamora-Camacho FJ (2019) Integrating time progression in ecoimmunology studies: beyond immune response intensity. Curr Zool 65: 205–212. 10.1093/cz/zoy045. 30936910 PMC6430971

[ref138] Zamora-Camacho FJ , Comas M (2018) Early swelling response to phytohemagglutinin is lower in older toads. PeerJ 6: e6104. 10.7717/peerj.6104. 30595980 PMC6304268

[ref139] Zamora-Camacho FJ , Zambrano-Fernández S, Aragón P (2022) Long-term sex-dependent inflammatory response of adult frogs to ammonium exposure during the larval stage. Chemosphere 307: 136202. 10.1016/j.chemosphere.2022.136202. 36037957

[ref140] Zapata AG , Varas A, Torroba M (1992) Seasonal variations in the immune system of lower vertebrates. Immunol Today 13: 142–147. 10.1016/0167-5699(92)90112-K. 1580995

[ref141] Zhang Z , Jin C, Qu K, Caviedes-Vidal E (2017) Immune responsiveness to phytohemagglutinin displays species but not sex differences in three anuran species. PeerJ 5: e3181. 10.7717/peerj.3181. 28480133 PMC5419208

