# Supplementary material for: Critical review of the phytohemagglutinin assay for assessing amphibian immunity
Source: Conserv Physiol. 2023 Dec 12;11(1):coad090. doi: 10.1093/conphys/coad090 (PMC10714196; doi:10.1093/conphys/coad090)
Supplement: Web_Material_coad090 [file web_material_coad090.pdf]

SUPPLEMENTAL MATERIALS

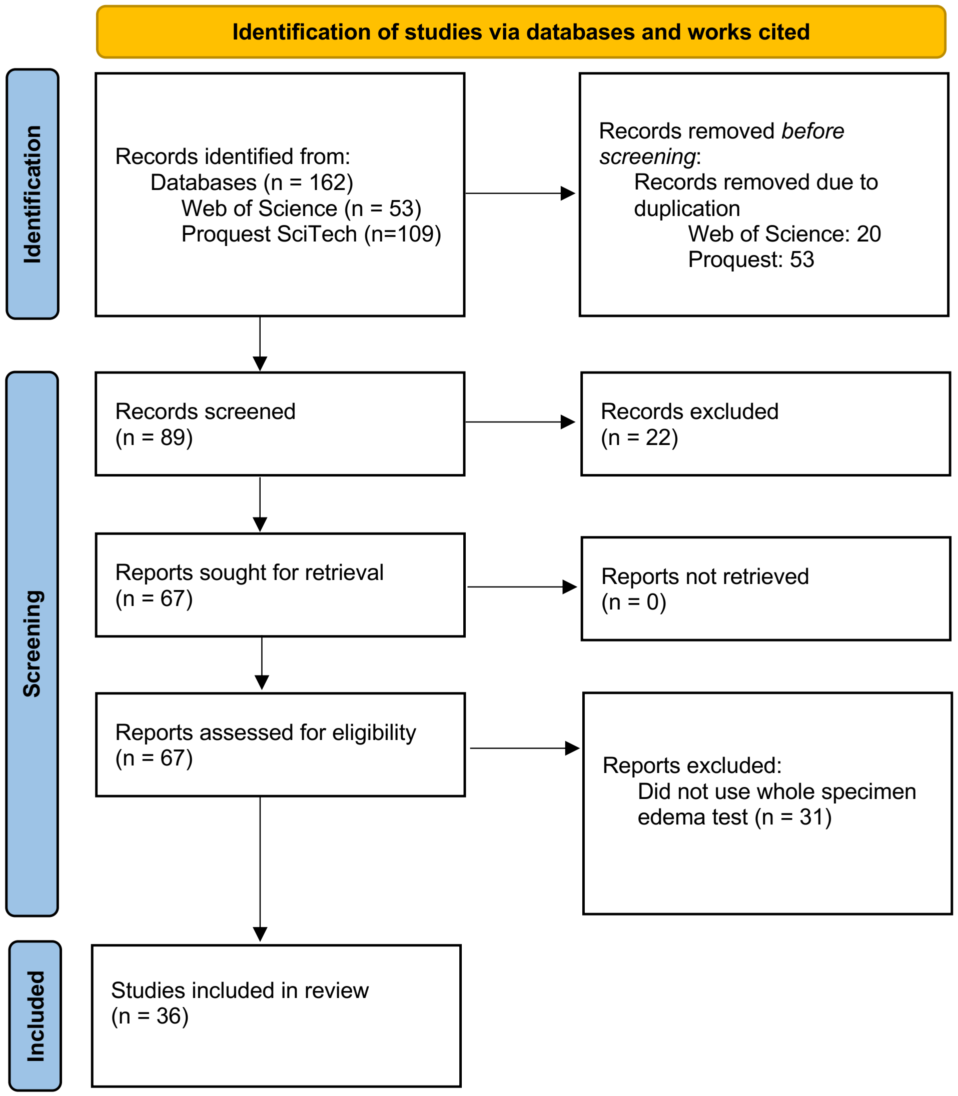

Supplementary Figure 1. Amphibian PHA Literature Data Collection and Methodology.

This review used the well-established PRISMA framework (Sarkis-Onofre *et al.*, 2021) to capture all relevant studies using the Web of Science and Proquest literature databases. The Web of Science search using amphibian search terms (((ALL=(phytohaemagglutinin)) OR ALL=(phytohemagglutinin)) OR ALL=(phytohemagglutination)) AND (((((ALL=(amphibian)) OR ALL=(frog)) OR ALL=(anuran)) OR ALL=(toad)) OR ALL=(salamander)) returned 53 results. A

duplicate search was made using Proquest, which uses differing query language syntax: noft((phytohaemagglutinin OR phytohemagglutinin OR phytohemagglutination)) AND noft((anuran* OR frog* OR toad* OR salamander* OR amphibian*)). This search returned 109 results. Duplicate and irrelevant sources were excluded, leaving 36 publications on the PHA assay in amphibians used in our review.

(a)

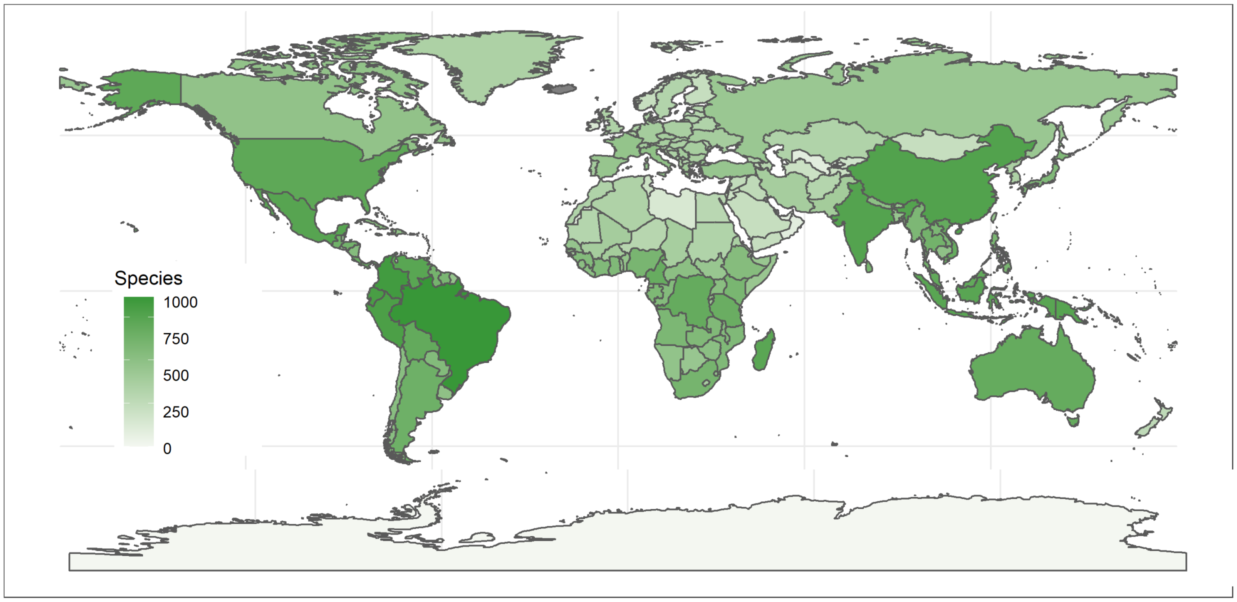

(b)

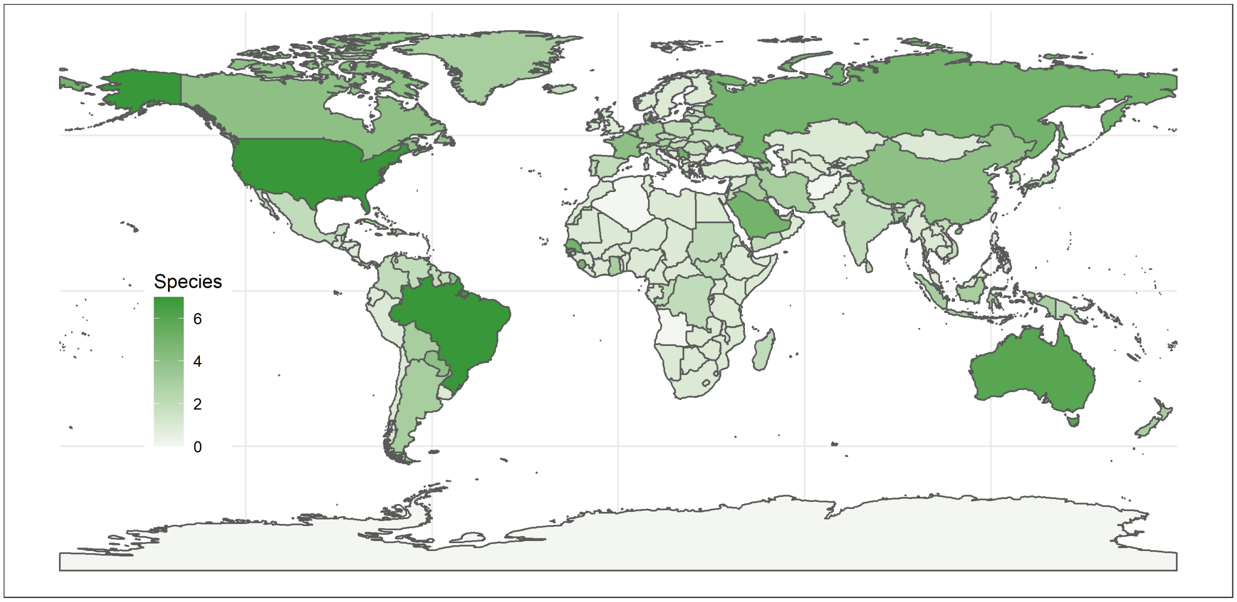

Supplementary Figure 2. Amphibian PHA Literature Data Collection and Methodology.

Global distribution of (a) all amphibian species and (b) amphibian species tested in PHA studies (see **Table 1** in main text).
